# Health outcomes following childhood or adolescent exposure to household food insecurity: a rapid systematic review

**DOI:** 10.1017/S1368980025101109

**Published:** 2025-09-19

**Authors:** Emily C. Clark, Erin Reyce, Sarah E. Neil-Sztramko, Valerie Tarasuk

**Affiliations:** 1 National Collaborating Centre for Methods and Tools, McMaster University, McMaster Innovation Park, 175 Longwood Rd S, Suite 210a, Hamilton ON L8P 0A1, Canada; 2 North Bay Parry Sound Health Unit, 345 Oak St W, North Bay ON P1B 2T2, Canada; 3 Department of Health Research Methods, Evidence & Impact, McMaster University, Health Sciences Centre 2C, 1280 Main St W, Hamilton ON L8S 4L8, Canada; 4 Department of Nutritional Sciences, University of Toronto, Medical Sciences Building Room 5253, 1 King’s College Circle, Toronto ON M5S 1A8, Canada

**Keywords:** Food security, Household food security, Health, Children, Youth, Mental health, Oral health

## Abstract

**Objectives:**

Household food insecurity (HFI) is a social determinant of health globally. Rates of HFI have risen in many high-income countries in recent years, particularly in households with children. The health outcomes associated with HFI for children and adolescents have not been systematically synthesised. This review was conducted to support advocacy efforts for meaningful policy action to reduce HFI in households with children.

**Design:**

A systematic search was conducted in Medline, Embase and PsycInfo databases. Primary studies measuring the association between physical or mental health outcomes and HFI were included. Studies were appraised and population, setting, measures and outcomes were extracted. Findings were grouped by related outcomes. Due to heterogeneity, findings were synthesised narratively. Rapid review methodology was used to accommodate resource constraints.

**Setting:**

High-income countries.

**Participants:**

Youth aged less than 18 years.

**Results:**

Thirty-six studies were included. Most were cross-sectional studies conducted in the USA. Outcomes included general health, early childhood, cardiometabolic, asthma, dental caries, mental health, sleep, diet and anaemia. Despite substantial heterogeneity in HFI measures and analysis, findings support associations between HFI and negative outcomes for general health status, asthma, dental caries and mental health. Findings for other outcomes were mixed.

**Conclusions:**

This review clarifies the effects of HFI on children and adolescents. Findings highlight trends for negative physical and mental health outcomes associated with HFI during youth, particularly related to mental health, oral health, asthma and general health status. Policy-level action should address rising rates of HFI and long-term effects on these vulnerable populations.

Food security is defined by the FAO of the UN as ‘a situation that exists when all people, at all times, have physical, social and economic access to sufficient, safe and nutritious food that meets their dietary needs and food preferences for an active and healthy life’^(1)^. Food security requires available, accessible, stable food sources that are utilised by individuals or households. Household food insecurity (HFI) is an ongoing and worsening public health problem in high-income countries around the world. In Canada, HFI is defined as the inability to acquire or consume a sufficient quantity or quality of food^(2)^. In the USA, the United Kingdom, Australia and Canada, HFI has been seen in rising rates in recent years^(3–6)^. Households with children are at particularly high risk of experiencing HFI^(4,5)^.

HFI is well understood globally as a social determinant of health with a strong association with household income^(7,8)^. Research evidence is mounting for the associations of negative health outcomes and HFI in high-income countries. In adulthood, cardiometabolic conditions linked to diet, such as diabetes, are associated with HFI^(9–15)^. HFI is associated with premature mortality^(16)^ and the inability to adhere to prescription medication^(17)^. People experiencing HFI are also more likely to require mental health care services^(17)^ and experience chronic pain requiring the use of opioids^(18)^. Evidence shows that HFI is linked to increased healthcare costs, exacerbating strains on already over-burdened health systems^(19–25)^.

Research also points to negative health outcomes associated with HFI experienced during childhood and adolescence, but the long-term impact of HFI during this developmentally sensitive period is not fully understood. There is some evidence examining the impact of HFI on early childhood development and physiological outcomes in children aged 0–5 years, demonstrating that HFI is associated with developmental risks and numerous negative physical health outcomes such as anaemia and chronic illness^(26,27)^. In later years, there is evidence that the mental health of youth may be particularly affected by HFI, with reports of increased depression and other mental health issues^(28,29)^. Similar to adults, evidence shows that youth experiencing HFI use health services more frequently and incur higher healthcare costs^(30,31)^.

Despite the increased risk of HFI in households with children and youth and evidence for its detrimental effects on physical and mental health, a comprehensive synthesis capturing the full breadth of health outcomes is not currently available. This rapid systematic review aims to address this gap and synthesise what is known about the association between exposure to HFI during childhood and/or adolescence in high-income countries and physical and mental health outcomes, using studies with stringent measures of HFI. Understanding the full scope of this issue is critical to support advocacy efforts to increase awareness and influence decision makers to take meaningful policy action to reduce HFI among households with children and youth.

## Methods

### Study design

This rapid review was completed in collaboration with Canada’s National Collaborating Centre for Methods and Tools’ Rapid Evidence Service^(32)^. The review was not registered. The review was conducted and reported according to the Preferred Reporting Items for Systematic Reviews and Meta-Analyses guidelines, with deviations to accommodate the rapid timelines reported below^(33)^. A rapid review approach was used given external time and resource constraints and to allow for review completion during a finite professional development placement opportunity. Previous research has demonstrated that rapid reviews report similar conclusions to full systematic reviews, though often with fewer details^(34)^.

### Information sources and search strategy

A public health librarian supported the development of the search strategy. The search strategy underwent peer review through Ontario’s public health Shared Library Services Partnership^(35)^. The search was conducted in Medline, Embase and PsycInfo databases on October 4, 2023, using terms related to ‘food security’, ‘child’ and ‘youth’. To ensure broad health outcomes were captured by the search, the search was not restricted by terms related to outcomes. The full search strategy is included in Appendix A.

Following de-duplication, all identified references were screened using DistillerSR software, first by title and abstract and then by full text. For title and abstract screening, two independent reviewers screened of a sample of ∼10 % of retrieved references. As > 85 % agreement was achieved, the remaining screening was completed by a single reviewer, consistent with a rapid review timeline. The DistillerSR artificial intelligence re-rank function was used to continually assign predicted relevance scores to references and sort references by highest predicted relevance score. References with a relevance score < 0·8 were automatically excluded and not reviewed by a human reviewer^(36)^. Full-text articles deemed potentially relevant at the title and abstract level were screened for eligibility by a single reviewer and double checked by a second reviewer.

### Eligibility criteria

English-language primary studies using a quantitative methodology to assess the relationship between experiencing HFI and a physical or mental health outcome were eligible for inclusion. Grey literature was not included to allow for a rapid review timeline. Systematic reviews were excluded to allow for data extraction directly from primary studies^(37)^. Eligible studies included infants, toddlers, children and adolescents aged 18 years and under. To manage the scope of the review, studies focused on maternal health, including studies of HFI during pregnancy and breastfeeding, were excluded. To maximise the generalisability of review findings, studies focused on children or adolescents with chronic health issues (e.g. examining the effects of HFI on a subset of youth living with existing health conditions, such as renal disease or cystic fibrosis) were also excluded. Across the literature, there is high variability in the methods used to measure HFI across studies^(38)^. The most well-established tool for measuring HFI is the United States Department of Agriculture’s 18-item Household Food Security Survey Module (HFSSM)^(39)^. This tool has been adapted for use in other national surveys, such as in Canada and South Korea^(40,41)^, and with specific populations^(42,43)^. Many studies, however, use 1- or 2-question survey items to determine HFI in participants^(26,44)^. While some advocate for the use of these shorter surveys to reduce respondent burden, questions regarding their sensitivity remain^(45,46)^. Studies that measured HFI using a validated survey tool and at least four questions to establish the level of HFI were included^(47)^. HFI measured as a binary, e.g. household food insecure *v*. household food secure, or as a gradient, e.g. severe, moderate, marginal or no food insecurity, was eligible for inclusion. Any physical or mental health-related outcome was eligible for inclusion; however, studies that only measured outcomes related to body size, such as BMI and overweight/obesity, were excluded, given the need in public health practice to divest from using weight-related outcomes as indicators of health^(48)^. Studies that only examined early childhood development, academic and behaviour-related outcomes were also excluded. Studies conducted in low- and middle-income countries were excluded^(49)^.

### Quality assessment

The quality of the included studies was evaluated using the JBI suite of critical appraisal tools^(50)^. According to the critical appraisal results, studies were rated low, moderate or high quality at pre-defined threshold scores. Two reviewers completed the quality assessment independently, and conflicts were resolved through discussion.

### Data extraction

Two members of the research team completed data extraction for selected articles independently, and conflicts were resolved through discussion. Data for study design, location, population and outcomes were extracted. Data regarding the measurement of HFI, including the survey tool used, the source of data, thresholds to determine levels of HFI and co-variates used in the analysis, were also extracted. Findings with *P* < 0·05 were considered statistically significant and extracted; non-significant findings were not recorded in the data table to expedite the rapid review timeline. Given the anticipated heterogeneity of covariates used to conduct analyses in each study, both adjusted and unadjusted estimates of effect were extracted.

### Data analysis

Meta-analysis was not planned due to the expected heterogeneity of study outcomes and HFI measures. The health outcomes examined in relation to HFI in the included studies were grouped according to nine categories. Findings of studies that reported on outcomes relevant to more than one category were included in more than one analysis. These categories were general health status (e.g. self or parent ratings of general health, chronic health conditions, use of health services and cortisol levels), early childhood (e.g. infant mortality, failure to thrive, growth, development and caregiver-rated overall health), cardiometabolic health (e.g. blood pressure, cholesterol levels and diabetes or HbA1c levels), asthma, dental caries, mental health (e.g. mental health disorders and symptoms and self-esteem), sleep, dietary outcomes (e.g. calorie, macro- and micronutrient intake) and anaemia. Study findings were summarised descriptively and synthesised narratively. Study quality, sample size and study design were considered in the synthesis and interpretation of the findings. Findings are summarised according to reported analyses, including whether there was a statistically significant association between the outcome(s) of interest and HFI as a binary variable, gradients associated with increased severity of HFI and associations with different threshold levels of HFI.

## Results

**Figure 1.**
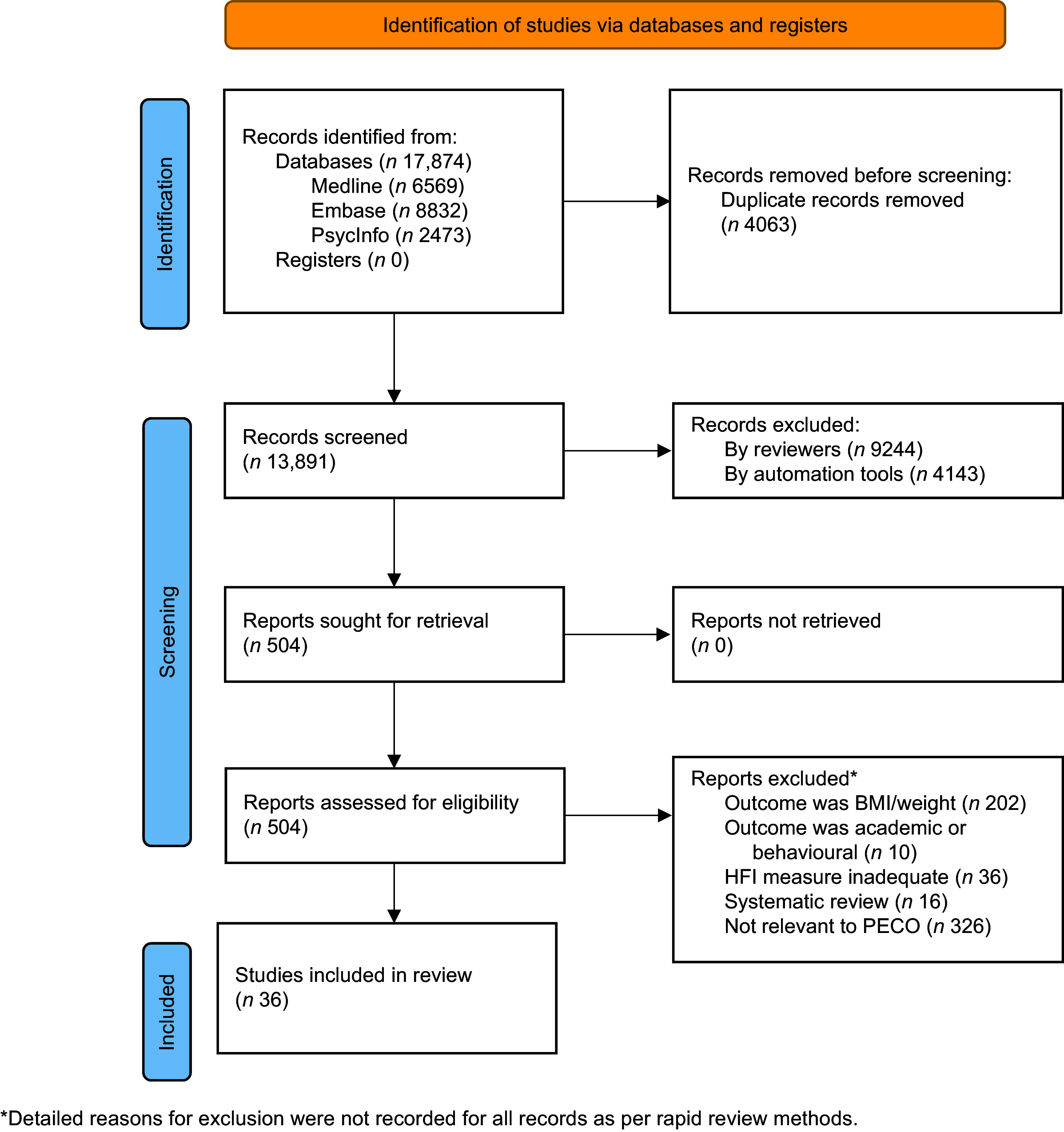
PRISMA 2020 flow chart.

A total of 17 874 records were retrieved after database searching. After removing duplicates, 9748 records were screened by title and abstract, 5605 by a human reviewer and 4143 by AI. The remaining 504 records were assessed at the full-text level by a human review, with thirty-six of these articles deemed eligible for inclusion (Figure 1). Consistent with a rapid review methodology, the reason for exclusion at the full-text level was not recorded for all studies.

### Study characteristics

Of the thirty-six included studies, most (*n* 27, 75 %) were conducted in the United States, followed by Canada (*n* 7, 19 %)^(30,51–56)^ and one study each from France^(57)^ and South Korea^(58)^ (Table 1). Most studies (*n* 31, 86 %) included a general or low-income sample of children, while one focused on Urban American Indian/Alaska Native children^(69)^, one on Latinx children^(66)^ and one on Inuit children^(51)^. Study samples had a wide range of proportions experiencing HFI, from 5·1 % to 86·7 %.

**Table 1.** Included studies of HFI, grouped by outcomes

Study	Study design; quality rating	Study sample	Setting/data source and location	HFI measure and threshold(s)^*^	Outcomes	Covariates	Findings (unadjusted)	Findings (adjusted)
General health status								
Jung 2023^(58)^	Cross sectional; low quality	*n* 3555; aged 10–18 years *n* 405 (11·4 %) food insecure	Korea National Health and Nutrition Examination Survey (KNHANES) 2012–2019 South Korea	18-item KNHANES survey adapted from 18-item HFSSM 0–2: FS 3–7: mild FI 8–12: moderate FI ≥ 13: severe FI	Overall health, stress levels	None	Results were presented separately for boys and girls. For any level of HFI, -In girls and boys there were higher rates of reported fair/poor health (*P* < 0·001 girls, *P* = 0·005 boys) -In girls, there were higher stress levels (*P* = 0·028). Findings for stress levels were non-significant in boys.	NR
Men 2021^(52)^	Cohort; moderate quality	*n* 212 300; aged 12–17 years *n* 18 600 (11 %) food insecure	Canadian Community Health Survey 2005–2017 Ontario and Alberta, Canada	8-item HFSSM 0: FS 1: marginal FI 2–4: moderate FI ≥ 5: severe FI	Injury-related emergency department visits in past 12 months	Child: sex, age, race/ethnicity, immigrant status, tobacco use, alcohol consumption, emergency department visits in previous year Caregiver: education Household: income, rent/own home, household type (couple with children, couple without children, lone parents, others)	Children with any level of HFI had greater incident rate ratio of injury-related emergency visits, -Marginal FI IRR = 1·19, 95 % CI = 1·10, 1·29 -Moderate FI IRR = 1·44, 95 % CI = 1·34, 1·54 -Severe FI IRR = 1·87, 95 % CI = 1·72, 2·04	Children with moderate or severe HFI had greater incident rate ratio of injury-related emergency visits, -Moderate FI IRR = 1·16, 95 % CI = 1·07, 1·25 -Severe FI IRR = 1·35, 95 % CI = 1·24, 1·48
Pirkle 2014^(51)^	Cross-sectional; high quality	*n* 292; aged 8·5–14·3 years *n* 145 (49·7 %) food insecure	Cord Blood Monitoring Program 1993–1998, or Environmental Contaminants and Infant Development Study 1996–2000 Nunavik, Northern Québec, Canada	4-item HFSSM adapted to Nunavik context ≥ 1: FI	Stature	Child: sex, age, body size, prenatal lead exposure, and postnatal polychlorinated biphenyls exposure	Children with HFI were 2·41 cm shorter (95 % CI = –0·97, –3·95).	There was no statistically significant difference in stature for children with or without HFI.
Sandoval 2021^(59)^	Cohort; high quality	*n* 268; aged 0–18 months *n* 32 (13·6 %)	Academically-affiliated safety net medical centre 2018–2019 Los Angeles, California, USA	6-item HFSSM ≥ 1: FI	Use of health services	Caregiver: race/ethnicity, education, employment status, preferred language, number of prior gestations and prior live births, relationship with child’s father, Household: presence of father in the home	NR	Children with HFI had a higher incident rate ratio of inpatient visits (IRR = 2·4, *P* = 0·04)
Tarullo 2020^(60)^	Cross-sectional; high quality	*n* 173; aged 1 or 3·5 years Proportion food insecure NR	Data collection dates NR Boston, USA	9-item Household Food Insecurity Access scale (HFIAS) Threshold not specified	Hair and salivary cortisol levels	Caregiver: hair cortisol levels, sensitivity	NR	Children with HFI had: -Higher hair cortisol concentration at 1- and 3·5 years (values NR; *P* < 0·05) -Flattened diurnal cortisol slope at 3·5 years (*P* < 0·05)
Thomas 2019^(61)^	Cross-sectional; high quality	*n* 29 341; aged 2–17 years *n* 4080 (13·9 %) food insecure	National Health Interview Survey 2013–2016 USA	10-item USDA 30-day food security measure ≥ 3: FI	General health status, chronic health conditions, use of acute health services	Child: sex, age, race/ethnicity, disability, citizenship, health insurance status Caregiver: perceived health status, weekly work hours, cigarette and alcohol use Household: income, region of residence, average age of adults, parent in home, number of children in home, social assistance status, rent or own, neighbourhood quality index	NR	Children with HFI (% difference, all *P* < 0·05): -Rated lower perceived general health status (2·5 % more frequently) -Had more acute care visits (25·9 %) -Were more likely to have chronic health conditions, including past asthma diagnosis (16·3 %), current asthma (19·1 %), eczema or other skin allergies (49·3 %) -Were more likely to have had a cold in past 2 weeks (21·8 %) -Were more likely to have had stomach problems (41·2 %)
Early childhood health								
Cassidy-Vu 2022^(62)^	Cross-sectional; high quality	*n* = data from 100 counties (number of individuals NR) *n* 15·4 % food insecure	County Health Rankings 2019 North Carolina, USA	18-item HFSSM ≥ 3: FI	Infant mortality	County level population size, race/ethnicity, % uninsured, education levels, household income, health status (diabetes and obesity), health behaviours (smoking, excessive drinking, physical inactivity), health outcomes (teenage pregnancy, low birthweight)	NR	Of all variables tested, HFI was the strongest statistically significant predictor variable of infant mortality rates.
Drennan 2019^(63)^	Cross-sectional, moderate quality	*n* 28 184; aged 0–4 years *n* 14 % food insecure	Children’s HealthWatch Survey 2009–2017 USA	18-item HFSSM ≥ 3: FI	Growth, development, parent-rated overall health	Child: age, birth weight Caregiver: race/ethnicity maternal age, education, marital status, employment, food assistance program participation	NR	Children with HFI had increased odds of reporting fair/poor health (aOR = 1·42, 95 % CI = 1·18, 1·71). Findings for growth and development were not statistically significant.
Edwards 2023^(64)^	Cross-sectional, high quality	*n* 7356; aged 0–3 years *n* 38·5 % food insecure	National Health and Nutrition Examination Survey (NHANES) USA	18-item HFSSM Thresholds NR	Underweight (failure to thrive)	Caregiver: race/ethnicity Household: income	There were no significant differences between the odds of underweight for children with and without HFI.	There were no significant differences between the odds of underweight for children with and without HFI.
Cardiovascular health								
Jung 2023^(58)^	Cross sectional; low quality	*n* 3555; aged 10–18 years *n* 405 (11·4 %) food insecure	KNHANES 2012–2019 South Korea	KNHANES adapted from 18-item HFSSM 0–2: FS 3–7: mild FI 8–12: moderate FI ≥ 13: severe FI	Blood levels of HbA1c, fasting blood sugar, total cholesterol, HDL, LDL, TAG	None	Results were presented separately for boys and girls. For any level of HFI, -In girls, blood HDL levels were lower (*P* = 0·0003) -In boys, total cholesterol was lower (*P* = 0·0003) Findings for HbA1c, fasting blood sugar, blood LDL and TAG levels were not statistically significant.	NR
Fulay 2022^(65)^	Cross-sectional, high quality	*n* 2876; aged 12–17 years in low-income households *n* 1560 (54·2 %) food insecure	NHANES 2007–2016 USA	18-item HFSSM 0: FS 1–2: marginal FI 3–7: moderate FI ≥ 8: severe FI	Blood pressure, fasting blood sugar, total cholesterol, HDL, LDL, TAG	Child: sex, age, race/ethnicity, activity levels, sedentary behaviours, smoking Caregiver: education, marital status Household: income	NR	There were no significant differences in outcomes for children with and without HFI.
Clemens 2021^(53)^	Cohort; high quality	*n* 34 042; aged 0–17 years *n* 5·3 % food insecure	Canadian Community Health Survey 2005–2018 Canada	8-item child scale in HFSSM 0–1: FS 2–4: moderate FI ≥ 5: severe FI	Incident diabetes	Child: sex, age, race/ethnicity, comorbidities, location of residence, healthcare utilisation in previous year Caregiver: maternal age, education, Comorbidities, pregnancy complications, immigration status Household: income, smoking in home, own/rent home, number of children in home, single parent	For any level of HFI, increased risk of incident diabetes (HR = 1·69; 95 % CI = 1·01, 2·81)	There were no significant differences in incident diabetes for children with and without HFI.
Maldonado 2022^(66)^	Cross-sectional, high quality	*n* 1325; aged 8–16 years *n* 771 (58·2 %) food insecure	Hispanic Community Children’s Health Study/Study of Latino Youth 2012–2014 Bronx, New York; Chicago, Illinois; Miami, Florida; and San Diego, California, USA	18-item HFSSM 0: FS 1–2: marginal FI 3–7: moderate FI ≥ 8: severe FI	Blood pressure, fasting blood sugar, total cholesterol, HDL, LDL, TAG	Child: sex, age Caregiver: education, place of birth (USA or foreign) Household: income, location, food assistance	Children experiencing moderate or severe HFI had: -Lower HDL (–3·17 mg/dl, 95 % CI = –5·65, –0·70) -Higher TAG (8·68 mg/dl, 95 % CI = 1·75, 15·61) -Higher fasting blood sugar (1·37 mg/dl, 95 % CI = 0·08, 2·65) Findings for blood pressure, total cholesterol, and LDL were not statistically significant.	There were no significant differences in cardiometabolic markers for children with and without HFI.
Tester 2016^(67)^	Cross-sectional, high quality	*n* 1072; aged 12–18 years *n* 560 (52·2 %) food insecure	NHANES 2003–2010 USA	18-item HFSSM 0: FS 1–2: marginal FI 3–7: moderate FI ≥ 8: severe FI	Total cholesterol, HDL, LDL, TAG, apo B	Child: sex, age, race/ethnicity, BMI, waist:height ratio Caregiver: marital status, maternal education Household: income	NR	Children experiencing marginal HFI had: -Higher odds of elevated TAG ≥ 90 mg/dl (OR = 1·86, 95 % CI = 1·14, 3·05). -Higher odds of elevated TAG:HDL ratio (OR = 1·74, 95 % CI = 1·09, 2·78) -Higher odds of elevated apo B ≥ 90 mg/dl (OR = 1·98, 95 % CI = 1·17, 3·36) These were not seen for children with moderate or severe HFI. Findings for total cholesterol, LDL and apo B were not statistically significant.
Lee 2019^(68)^	Cross-sectional, high quality	*n* 2662; aged 12–19 years *n* 685 (18·4 %) food insecure	NHANES 2003–2014 USA	18-item HFSSM ≥ 1: HFI	Blood pressure, prediabetes (HbA1c 5·7–6·4 %)	Child: sex, age, race/ethnicity Household: income	NR	Children experiencing any level of HFI had: -Higher odds of prediabetes or diabetes (OR = 1·96, 95 % CI = 1·17, 3·19) -Higher odds of elevated blood pressure (OR = 1·65, 95 % CI = 1·38, 1·98)
Dong 2023^(69)^	Cross-sectional, high quality	*n* 142, aged 12–16 years *n* 78 (54·9 %) food insecure	Native American Youth Sleep Health and Wellness study 2018–2020 California, USA	9-item HFSSM 0: FS 1: marginal FI 2–5: moderate FI ≥ 6: severe FI	Blood pressure, cholesterol, HbA1c	Child: sex, age, race/ethnicity Household: single parent, financial hardship	NR	Children experiencing any level of HFI had: -Higher blood pressure (*P* = 0·03) Findings for cholesterol and HbA1c were not statistically significant.
South 2019^(70)^	Cross-sectional, high quality	*n* 7125, aged 8–17 years *n* 1446 (20·3 %) food insecure	NHANES 2007–2014 USA	18-item HFSSM 0: FS 1–2: marginal FI 3–7: moderate FI ≥ 8: severe FI	Blood pressure	Child: sex, age, race/ethnicity Household: income	NR	Children experiencing any level of HFI had: -Higher odds of elevated blood pressure (OR = 1·26, 95 % CI = 1·04, 1·54)
Asthma								
Mangini 2015^(71)^	Cohort; high quality	*n* 11 099 in grade 3 (ages NR) *n* 760 (6·8 %) food insecure	Early Childhood Longitudinal Study-Kindergarten 1998–2007 |USA	18-item HFSSM 0: FS 1–2: marginal FI ≥ 3: HFI	Asthma	Child: sex, race/ethnicity, height, weight, birthweight Caregiver: foreign born, depression Household: income, health insurance status	NR	Children experiencing any level of HFI had: -Higher odds of asthma (OR = 1·04, 95 % CI = 1·02, 1·06)
Thomas 2019^(61)^	Cross-sectional; high quality	*n* 29 341; aged 2–17 years *n* 4080 (13·9 %) food insecure	National Health Interview Survey 2013–2016 USA	10-item USDA 30-day food security measure ≥ 3: FI	Asthma	Child: sex, age, race/ethnicity, disability, citizenship, health insurance status Caregiver: perceived health status, weekly work hours, cigarette and alcohol use Household: income, region of residence, average age of adults, parent in home, number of children in home, social assistance status, rent or own, neighbourhood quality index	NR	Children with HFI (% difference, all *P* < 0·05): -Were more likely to have past asthma diagnosis (16·3 %) or current asthma (19·1 %).
Clemens 2021^(54)^	Cohort; high quality	*n* 27 746; aged 0–17 years *n* 5·1 % food insecure	Canadian Community Health Survey 2005–2018 Canada	8-item child scale in HFSSM 0–1: FS 2–4: moderate FI ≥ 5: severe FI	Incident asthma	Child: sex, age, race/ethnicity, comorbidities, history of prematurity or intrauterine growth restriction, healthcare utilisation in previous year Caregiver, maternal age, immigration status Household: income, smoking in home, own/rent home, number of children in home, single parent	NR	There were no significant differences in incident asthma for children with and without HFI.
Mangini 2018^(72)^	Cross-sectional, moderate quality	*n* 6731; Kindergarten to grade 8 (ages NR) *n* 467 (6·9 %) food insecure prior to Kindergarten *n* 385 (5·7 %) food insecure prior to grade 3	Early Childhood Longitudinal Study-Kindergarten 1998–2007 USA	18-item HFSSM ≥ 3: HFI	Asthma in Kindergarten and grade 2; incident asthma in grade 5 and grade 8	Child: sex, race/ethnicity, BMI Caregiver: foreign born, depression Household: income, health insurance status	NR	Children experiencing any level of HFI had: -Higher odds of asthma in Kindergarten (OR = 1·18, 95 % CI = 1·17, 1·20) -Higher odds of asthma in grade 2 (OR = 1·55, 95 % CI = 1·51, 1·55) -Higher odds of incident asthma in grades 5 and 8 (OR = 1·55, 95 % CI = 1·53, 1·58)
Dental caries								
Angelopoulou 2019^(73)^	Cross-sectional, moderate quality	*n* 82; aged 0–5 years *n* 34 (41·5 %) food insecure	Community dental clinic 2016 Milwaukee, Wisconsin, USA	6-item version HFSSM 0: FS 1: marginal FI 2–4: moderate FI ≥ 5: severe FI	Dental caries	None	HFI was associated with number of decayed, missing or filled teeth (*P* = 0·002). A gradient effect for decayed, missing or filled teeth was found, where prevalence increased with level of HFI (statistical significance NR)	NR
Bahanan 2021^(74)^	Cross-sectional, high quality	*n* 4822; aged 5–17 years Proportion food insecure NR	NHANES 2011–2012; 2013–2014 USA	18-item HFSSM 0: FS 1–2: marginal FI 3–7: moderate FI ≥ 8: severe FI	Untreated dental caries	Child: sex, age, race/ethnicity, oral health visits in last year, number of teeth Household: income, size, social assistance	NR	Children with any level of HFI had greater odds of untreated caries (OR = 1·38, 95 % CI = 1·11, 1·72)
Hill 2020^(75)^	Cross-sectional, moderate quality	*n* 4406; aged 1–19 years *n* 914 (20·7 %) food insecure	NHANES 2013–2014 USA	Assessment of child food security status 0: FS 1–2: marginal FI 3–7: moderate FI ≥ 8: severe FI	Dental caries	Child: age, race/ethnicity Household: income, social assistance	Children with any level of HFI had greater odds of dental caries (OR = 3·51, 95 % CI = 1·71, 7·19)	Children with severe HFI had greater odds of dental caries (OR = 2·84, 95 % CI = 1·13, 7·12). This association was not present for marginal or moderate HFI.
Mental health								
Anderson 2023^(30)^	Cross-sectional; high quality	*n* 32 321; aged 1–17 years *n* 5216 (16·1 %) food insecure	Canadian Community Health Survey 2005–2014 Ontario, Canada	8-item HFSSM 0: FS 1: marginal FS 2–5: low FS ≥ 6: very low FS	Use of health services for mental health or substance use	Child: sex, age, racial group Caregiver: maternal migrant status, past year maternal health service use for mental or substance use disorders, single parent status, Household: income, rurality, number of children	Children with any level of HFI had: -More outpatient visits for mental or substance use disorders in previous years (PR = 1·60, 95 % CI = 1·47, 1·74) -More acute care visits (PR = 1·71, 95 % CI = 1·23, 2·37) -More visits of any type for mental disorders (PR = 1·39, 95 % CI = 1·23, 1·58), substance use disorders (PR = 1·90, 95 % CI = 1·05, 3·43) and neurodevelopmental disorders (PR = 1·87, 95 % CI = 1·66, 2·10) A gradient effect for outpatient visits for mental or substance use disorders was found, where prevalence increased with level of HFI (test for trend PR = 1·25, 95 % CI = 1·21, 1·31)	When findings were partially adjusted for age, sex, income and rurality, children with any level of HFI had: -More outpatient visits for mental or substance use disorders in previous years (PR = 1·55, 95 % CI = 1·41, 1·70) -More acute care visits (PR = 1·74, 95 % CI = 1·24, 2·45) -More visits of any type for mental disorders (PR = 1·43, 95 % CI = 1·25, 1·64), substance use disorders (PR = 2·26, 95 % CI = 1·18, 4·33) and neurodevelopmental disorders (PR = 1·71, 95 % CI = 1·50, 1·95) When findings were adjusted for all covariates, children with any level of HFI had: -More outpatient visits for mental or substance use disorders in previous years (PR = 1·39, 95 % CI = 1·26, 1·53) -More visits of any type for mental disorders (PR = 1·27, 95 % CI = 1·11, 1·46), and neurodevelopmental disorders (PR = 1·55, 95 % CI = 1·36, 1·77)
Burke 2016^(76)^	Cross-sectional; high quality	*n* 31 061; aged 4–17 years *n* 7678 (24·7 %) food insecure	National Health Interview Survey 2011–2014 USA	10-item HFSSM 0: FS 1–2: marginal FS 3–5: low FS ≥ 6: very low FS	Mental health disorders, including anxiety, mood, oppositional defiant, attention-deficit hyperactivity, and conduct disorders	Child: sex, age Caregiver: sex, age, race/ethnicity, education level, perceived health status Household: income, one *v*. two caregivers	NR	The odds of mental disorders increased with severity of food insecurity: For ages 4–11, -Marginal FS OR = 1·26, 95 % CI = 1·05, 1·52 -Low FS OR = 1·51, 95 % CI = 1·24, 1·82 -Very low FS OR = 1·59 (1·27, 2·00) For ages 12–17, -Marginal FS OR = 1·33, 95 % CI = 1·05, 1·68 -Low FS OR = 1·82, 95 % CI = 1·43, 2·34 -Very low FS OR = 2·55 (1·83, 3·57)
Godrich 2019^(55)^	Cross-sectional; moderate quality	*n* 5281; aged 10–11 years *n* 1331 (25·2 %) food insecure	Canadian Children’s Lifestyle and School Performance Study II 2011 Nova Scotia, Canada	6-item HFSSM 0: FS 1: marginal FI 2–4: moderate FI 5–6: severe FI	Self-esteem measured by 3-point Likert scale Self-efficacy measured by 9-item questionnaire	Child: sex, body size Caregiver: education Household: income, rurality	Children with any level of HFI had greater odds of low self-esteem, -Marginal FI OR = 1·49, 95 % CI = 1·10, 2·03 -Moderate FI OR = 1·65, 95 % CI = 1·25, 2·18 -Severe FI OR = 1·62, 95 % CI = 1·19, 2·20 Children with moderate or severe HFI had statistically significant lower self-efficacy to make healthy choices, -Moderate FI regression coefficient = –2·73, 95 % CI = –4·19, –1·27 -Severe FI regression coefficient = –2·27, 95 % CI = –4·04, –0·48	There were no statistically significant findings between HFI and self-esteem or self-efficacy after covariate adjustment.
Jung 2023^(58)^	Cross sectional; low quality	*n* 3555; aged 10–18 years *n* 405 (11·4 %) food insecure	KNHANES 2012–2019 South Korea	KNHANES adapted from 18-item HFSSM 0–2: FS 3–7: mild FI 8–12: moderate FI ≥ 13: severe FI	Depressive mood for ≥ 2 consecutive weeks, suicidal ideation in past year	None	Results are presented separately for boys and girls. Children with any level of HFI, -Girls had higher rate of depressive mood for ≥ 2 weeks, value NR, *P* < 0·002 Findings for suicidal ideation were not statistically significant.	NR
Maynard 2019^(77)^	Cross-sectional; high quality	*n* 935; aged 12–17 years *n* 290 (31 %) food insecure	NHANES 2009–2010 USA	18-item HFSSM ≥ 1: FI	Anxiety assessed with 1-item from Current Health Status Questionnaire	Child: sex, age, race/ethnicity Caregiver: education Household: income, size of household	NR	Children with any level of HFI had greater odds of experiencing anxiety (aOR = 1·64, 95 % CI = 1·04, 2·60)
Poole-Di Salvo 2016^(78)^	Cross-sectional; high quality	*n* 8600; aged 12–16 years *n* 877 (10·2 %) food insecure	Early Childhood Longitudinal Study 2007 USA	18-item HFSSM ≥ 3: FI	Mental health assessed with parent-reported Strengths and Difficulties Questionnaire	Child: sex, age, grade level, race/ethnicity, BMI, stressful life events Caregiver: perceived health status, maternal age, marital status Household: income, perceived neighbourhood safety	NR	Children with any level of HFI had great odds of: -mental health issues (aOR = 2·30, 95 % CI = 1·62, 3·27) -emotional misconduct (aOR = 2·49, 95 % CI = 1·65, 3·75) -peer problems (aOR = 1·58, 95 % CI = 1·02, 2·45) -less optimal pro-social behaviour (aOR = 1·55, 95 % CI = 1·05, 2·23)
Sleep								
Dong 2023^(69)^	Cross-sectional, high quality	*n* 142; aged 12–16 years *n* 78 (54·9 %) food insecure	Native American Youth Sleep Health and Wellness study 2018–2020 California, USA	9-item HFSSM 0: FS 1: marginal FI 2–5: moderate FI ≥ 6: severe FI	Sleep disturbance, measured by School Sleep Habits Survey for Adolescents, and actigraphy × 7 d	Child: sex, age, race/ethnicity Household: single parent, financial hardship	NR	Children experiencing any level of HFI had: -Greater sleep disturbance (*P* < 0·001)
Na 2020^(79)^	Cross-sectional, high quality	*n* 362; parents of preschool-aged children (ages NR) *n* 135 (37·3 %) food insecure	Headstart Preschool Classrooms 2017–2018 Pennsylvania, USA	18-item HFSSM ≥ 3: FI	Sleep quality	Child: sex, age Caregiver: age, education, marital status, employment status Household: number of children, size, income, social assistance, region, household chaos, family functioning	Children experiencing any level of HFI had: -Increased odds of poor sleep quality (OR = 1·65, 95 % CI = 1·07, 2·55)	There were no statistically significant findings between HFI and sleep quality.
Diet								
Jung 2023^(58)^	Cross sectional; low quality	*n* 3555; aged 10–18 years *n* 405 (11·4 %) food insecure	KNHANES 2012–2019 South Korea	KNHANES adapted from 18-item HFSSM 0–2: FS 3–7: mild FI 8–12: moderate FI ≥ 13: severe FI	Nutrient intake measured using 2010 Dietary Reference Intake for Koreans Frequency of meals, eating with family or other people, frequency of dining out, utilisation of nutrition facts, influence of nutrition facts	None	Results are presented separately for boys and girls. Children with any level of HFI, -Boys had lower vitamin A intake, value NR, *P* = 0·0002 -Girls had lower protein intake, value NR, *P* = 0·039 -Girls had lower niacin intake, value NR, *P* = 0·032 -Boys had higher rates of skipping breakfast, value NR, *P* = 0·039 -Girls had higher rates of skipping lunch, value NR, *P* = 0·028 Other findings were not statistically significant.	NR
Bahanan 2021^(74)^	Cross-sectional, high quality	*n* 4822; aged 5–17 years Proportion food insecure NR	NHANES 2011–2012; 2013–2014 USA	18-item HFSSM 0: FS 1–2: marginal FI 3–7: moderate FI ≥ 8: severe FI	Diet quality	Child: sex, age, race/ethnicity, oral health visits in last year, number of teeth Household: income, size, social assistance	NR	Children with any level of HFI had lower mean scores of whole fruits consumption (value NR, *P* = 0·01), seafood and plant protein consumption (value NR, *P* = 0·008). Other findings were not statistically significant.
Jun 2021^(80)^	Cross-sectional, moderate quality	*n* 9147; aged 1–18 years *n* 1226 (13·4 %) food insecure	NHANES 2011–2016 USA	18-item HFSSM Thresholds NR	Diet quality measured by Healthy Eating Index-2015, nutrient intake, biomarkers of nutritional status (vitamin D, folate, Fe and Zn)	Child: age, race/ethnicity, supplement use, federal nutrition program	NR	Children experiencing HFI had higher risk of micronutrient inadequacy, -For boys and girls: vitamin D, Zn, choline and Mg. -For girls: Ca (All *P* < 0·05) Other findings were not statistically significant.
Canter 2017^(81)^	Cross-sectional, moderate quality	*n* 148; aged 5–10 years Proportion food insecure NR	Study survey 2013 Midwest USA	18-item HFSSM Thresholds NR	Fruit and vegetable consumption	Child: sex, age Household: coupon program use	NR	Children experiencing HFI had fewer servings of vegetables (–0·18, *P* < 0·05)
Hutchinson 2022^(56)^	Cross-sectional, high quality	Number NR; aged 0–18 years	Canadian Community Health Survey-Nutrition 2015 Canada	8-item HFSSM 0: FS 1: marginal FI 2–5: moderate FI ≥ 6: severe FI	Diet quality measured by Canadian Healthy Eating Index	Child: sex, age Household: income, education, region	NR	When adjusted for child age and sex, children aged 2–8 years experiencing any level of HFI had: -Lower diet quality scores -Fewer vegetable and fruit servings -Higher proportion of energy from ultra processed foods -Lower proportion of energy from protein -Higher Na intake per 1000 kcal consumed -Lower vitamin A, vitamin D, riboflavin, vitamin B_6_, Ca, Mg, phosphorous and potassium intake All *P* < 0·05) When adjusted for child age and sex, children aged 9–18 years experiencing any level of HFI had: -Lower diet quality scores -Lower proportion of energy from protein -Lower vitamin A, riboflavin, vitamin B_12_, Ca, Mg, phosphorous and Zn intake (All *P* < 0·05) When adjusted for all covariates, children aged 1–8 experiencing moderate HFI had: -Lower diet quality scores -Lower fruit and vegetable intake All *P* < 0·05) A gradient effect for intake of some micronutrients found, where intake decreased with increasing level of HFI. Other findings were not statistically significant.
Lee 2019^(82)^	Cross-sectional, high quality	*n* 193; aged 8–12 years *n* 55 (15 %) food insecure	Healthy Home Offerings via the Mealtime Environment (HOME) Plus study 2011–2017 Minneapolis, Minnesota, USA	6-item HFSSM short form ≥ 2: FI	Diet quality during summer months measured by Healthy Eating Index-2015	Child: age, BMI-z Caregiver: education	NR	On weekend days, children experiencing any level of HFI in FI households reported: -Less energy intake -Fewer cups whole fruit per 1000 kcal -More sugar-sweetened beverages consumed per 1000 kcal (All *P* < 0·05) These differences were not seen on weekdays.
Rossen 2016^(83)^	Cross-sectional, high quality	*n* 5136; aged 2–15 years *n* 822 (16 %) food insecure	NHANES 2007–2010 USA	18-item HFSSM 0: FS 1: marginal FI 2–4: moderate FI ≥ 5: severe FI	Dietary intake	Child: race/ethnicity, insurance status, foreign-born Caregiver: age, education, marital status, Household: income, size, smoking, food assistance programs	There were no statistically significant findings between HFI and dietary intake.	There were no statistically significant findings between HFI and dietary intake.
Trude 2022^(84)^	Cross-sectional, high quality	*n* 451; aged 9–15 years *n* 307 (68·1 %) food insecure	B’more Healthy Communities for Kids study 2013–2014 Baltimore, Maryland, USA	18-item HFSSM 0: FS 1–2: marginal FI 3–7: moderate FI ≥ 8: severe FI	Dietary intake measured by the Block Kids 2004 Food Frequency Questionnaire	Child: sex, age, BMI Caregiver: education	NR	Children experiencing moderate or severe HFI had: - Increased daily calorie intake (100 kcal, *P* = 0·04) -Increased intake of added sugar (4 g, *P* < 0·01) -Decreased proportion of calories from protein (0·5 %, *P* = 0·03) Other findings were not statistically significant.
Anaemia								
Arnaud 2018^(57)^	Cross-sectional, high quality	*n* 673; aged 6 months-12 years *n* 583 (86·7 %)	Sheltered children experiencing houselessness 2013 Paris, France	18-item HFSSM For children aged 6 months-5 years, 0–1: FS 2–4: moderate FI ≥ 5: severe FI For children aged 6–12 years, 0–2: FS 3–7: FI without hunger 8–12: FI with moderate hunger ≥ 13: FI with severe hunger	Anaemia	Child: sex, age, breast-feeding duration (if age ≤ 5 years) dietary scores (if age 6–12 years) Caregiver: birthplace, French proficiency, shelter type, moderate-severe anaemia, duration of houselessness Household: income, cooking facilities	NR	Children aged 6 months to 5 years experiencing any level of HFI had higher prevalence of anaemia, -Moderate HFI: PR = 1·81 (95 % CI = 1·07, 3·07) -Severe HFI: PR = 2·56 (95 % CI = 1·13, 5·81) Children aged 6–12 experiencing FI with severe hunger: -Higher prevalence of moderate-to-severe anaemia (PR = 1·3, 95 % CI = 0. Children without cooking facilities were more likely to have moderate-to-severe anaemia (PR = 1·62, 95 % CI = 1·03, 2·56)
Metallinos-Katsaras 2016^(85)^	Cohort; high quality	*n* 17 381; aged 18 months *n* 3459 (19·9 %) food insecure	Massachusetts WIC Database 2001–2009 Massachusetts, USA	4-item HFSSM subscale Thresholds NR	Anaemia	Child: sex, age, race/ethnicity, breast-feeding Caregiver: education Household: size	NR	For children with low HFI, odds of anaemia were greater (OR = 1·42, 95 % CI = 1·27, 1·60). There were no statistically significant findings between very low HFI and anaemia.
Jun 2021^(80)^	Cross-sectional, moderate quality	*n* 9147; aged 1–18 years *n* 1226 (13·4 %) food insecure	NHANES 2011–2016 USA	18-item HFSSM Thresholds NR	Dietary Fe intake	Child: age, race/ethnicity, supplement use, federal nutrition program	NR	There were no statistically significant findings between HFI and dietary Fe intake.
Pirkle 2014^(51)^	Cross-sectional, high quality	*n* 292; aged 8·5–14·3 years *n* 145 (49·7 %) food insecure	Cord Blood Monitoring Program 1993–1998, or Environmental Contaminants and Infant Development Study 1996–2000 Nunavik, Northern Québec, Canada	4-item HFSSM adapted to Nunavik context ≥ 1: FI	Fe deficiency, anaemia	Child: sex, age, body size, prenatal lead exposure and postnatal polychlorinated biphenyls exposure	There were no statistically significant findings between HFI and Fe deficiency or anaemia.	There were no statistically significant findings between HFI and Fe deficiency or anaemia.

FI, food insecurity; FS, food security; HFI, household food insecurity; IRR, incident rate ratio; PR, prevalence ratio; USDA, United States Department of Agriculture; WIC, Special Supplemental Nutrition Program for Women, Infants and Children; NR, Not reported.

* Threshold refers to number of affirmative answers.

Six (17 %) included studies used a cohort design, of which two were conducted prospectively over nearly 10 years^(71,85)^, three conducted retrospective analyses of large datasets collected over 8–12 years^(52–54)^ and one conducted a retrospective analysis of birth outcomes in a 1-year time period^(59)^. Five cohort studies were rated as high quality^(53,54,59,71,85)^ and one as moderate quality^(52)^. Quality assessments are included in online supplementary material, Supplemental Table A2a. The remaining 30 (83 %) studies used a cross-sectional design, and twenty-two were rated as high quality^(30,51,56,57,60–62,64–70,74,76–79,82–84)^, seven as moderate quality^(55,63,72,73,75,80,81)^ and one as low quality^(58)^. Quality assessments are included in online supplementary material, Supplemental Table A2b. For most of these studies, methodological quality was enhanced by the use of large, population-level datasets, as well as validated tools for the measurement of HFI; common limitations included self-reported outcome measurements and some incomplete follow-up of study participants.

The most frequently used tool to measure HFI was the Household Food Security Survey Module (HFSSM). The HFSSM is a self-report measure of food access, availability and utilisation, with a primary focus on financial constraints over the last 12 months. The HFSSM was used in 23 (64 %) studies, and a version adapted to Korea was used in one study^(58)^. The remaining 12 (33 %) studies all used abbreviated versions of the HFSSM, ranging from four to ten items. Despite using the same tool, some studies stratified participants on a gradient by level of HFI (e.g. food secure, marginal, moderate and severe HFI), while others differentiated participants using a binary variable (food secure *v*. food insecure). The threshold definition of ‘food secure’ varied, sometimes defined as having zero, one or two affirmative responses to the HFSSM questions.

### Study findings

#### General health status

Overall, six mostly large, high-quality studies examining general health outcomes consistently found poorer outcomes for children experiencing HFI^(51,52,58–61)^. Two cross-sectional studies, a large high-quality study of children in the USA^(61)^ and a low-quality study of adolescents in South Korea^(58)^, found that youth experiencing HFI were more likely to rate their overall health as poor and were more likely to have chronic health conditions^(58,61)^. In unadjusted analyses of one small, high-quality cross-sectional study of Inuit children in Northern Quebec, Canada, of which nearly half reported HFI, those who experienced HFI were shorter in stature; however, this effect was not statistically significant when the analysis was adjusted for covariates^(51)^.

A large moderate-quality cohort study of adolescents in Canada found more emergency department visits for children with any degree of HFI. However, after adjusting for covariates, this effect was only present for moderate or severe HFI^(52)^. A smaller, high-quality cohort study looked at the use of health services for infants in the USA and found over twice as many inpatient visits among those experiencing HFI than those in food-secure households (incident rate ratio = 2·4, *P* = 0·04)^(59)^.

Two studies also reported on overall stress levels, as measured through self-report^(58)^ or hair or salivary cortisol levels^(60)^. In a low-quality cross-sectional study conducted in South Korea, self-reported stress was higher in adolescent girls in households experiencing HFI than adolescents in food-secure households^(58)^. A small, high-quality cross-sectional study of young children conducted in the USA found that children experiencing HFI had higher hair cortisol levels than food-secure peers^(60)^.

#### Early childhood outcomes

Findings of three included studies are mixed regarding the association between HFI and early childhood outcomes^(62–64)^. In one high-quality cross-sectional study using county-level data in North Carolina, USA, across all counties in the state, HFI prevalence was strongly associated with the infant mortality rate^(62)^. Conversely, in a second high-quality cross-sectional study that analysed data from the National Health and Nutrition Examination Survey (NHANES), no significant associations were found between HFI and the odds of failure to thrive for children^(64)^. Finally, a moderate-quality cross-sectional study using the Children’s HealthWatch Survey dataset found that caregivers of infants and children aged 0–4 years experiencing HFI had higher odds of poorer caregiver-rated health status compared with food-secure households, but no differences were found in child’s growth or development^(63)^.

#### Cardiometabolic outcomes

Findings for the relationship between HFI and blood pressure were mixed across five high-quality cross-sectional studies^(65,66,68–70)^. Two large studies of youth in the USA and one small study of American Indian youth found that any level of HFI was associated with higher odds of elevated blood pressure^(68–70)^. In contrast, a large study of youth in the USA and a smaller study of Hispanic youth found no associations^(65,66)^. The three studies that found an association between HFI and blood pressure all adjusted analyses for child age, sex, race/ethnicity and household income^(68,70)^. The two studies that did not find an association adjusted their analyses for additional covariates, such as activity levels, sedentary behaviours, caregiver education and place of birth and household food assistance participation^(65,66)^.

Findings across five cross-sectional studies were also mixed for the association between HFI and cholesterol^(58,65–67,69)^. In an analysis that did not adjust for any covariates, a large low-quality study of youth in Korea found that for any level of HFI, blood levels of HDL HDL were lower in girls, and total cholesterol was lower in boys compared with those in food secure households^(58)^. A small, high-quality study of youth in the USA found that marginal HFI was associated with higher odds of elevated TAG, TAG:HDL ratio and apo B, but values for those with moderate or severe HFI were not different from food secure households^(67)^. A study of Hispanic youth in the USA found that in unadjusted analyses, moderate or severe HFI was associated with lower HDL and higher TAG. However, this was not seen when the analysis was adjusted for covariates. Additionally, a large study of youth and a small study of American Indian youth in the USA rated high quality found no association between HFI and cholesterol levels^(65,69)^.

Similarly, findings for the association between type 2 diabetes and related markers and HFI were mixed. One large, high-quality cross-sectional study of youth in the USA found that any level of HFI was associated with higher odds of pre-diabetic HbA1c levels after adjusting for child age, sex, race/ethnicity and household income as covariates^(68)^. However, most studies found no association after adjusting for variables such as activity levels, sedentary behaviours, caregiver education and place of birth and single-parent households^(53,65,66,69)^ Three cross-sectional studies, including a large high-quality study in the USA^(65)^, a large low-quality study of youth in South Korea^(58)^ and a small high-quality study of American Indian youth in the USA, did not find an association between HFI and fasting blood sugar or HbA1c levels^(69)^. In unadjusted analyses, HFI was associated with incident diabetes in a large, high-quality cohort study of children in Canada and fasting blood sugar levels in a small, high-quality cross-sectional study of Hispanic youth in the USA; neither association was found when analyses were adjusted for covariates^(53,66)^. One study that found no association between HFI and diabetic markers did not adjust for any covariates^(58)^.

#### Asthma

Four studies found an association between HFI and asthma, including two studies of school-aged children that both used data from the Early Childhood Longitudinal Study high-quality cohort study^(71)^ and the other a moderate quality cross-sectional study^(72)^, and a very large high-quality cross-sectional study using NHANES data of children up to age 17^(61,71,72)^. However, a large, high-quality cohort study did not find an association between HFI and incident asthma^(54)^. Each study included many covariates in its analysis; the only study that did not find an association also included household smoking and children’s history of prematurity or intrauterine growth restriction as a covariate^(54)^.

#### Dental caries

Three cross-sectional studies found an association between HFI and dental caries^(73–75)^. These include two large studies using NHANES data in the USA, one of which one is high quality^(74)^ and one of moderate quality^(75)^; both noted significant associations when adjusting for covariates. The third was a small moderate quality study conducted in community dental centres in Wisconsin, USA, but did not adjust for covariates in the analysis^(73)^.

#### Mental health

Six cross-sectional studies, all using data from large, population-level datasets, explored the association between HFI and mental health outcomes, including anxiety, depressive mood, self-esteem and self-efficacy and use of health services of mental health or substance use^(30,55,58,76–78)^. Two high-quality studies from the USA explored the association between HFI and mental health issues broadly. Data from the 2011–2014 National Health Interview Survey found that the odds of mental disorders, including anxiety, mood, oppositional defiant, attention-deficit hyperactivity and conduct disorders, were higher for children aged 4–17 years with HFI compared with food-secure children^(76)^. The strength of the association increased with the severity of HFI^(76)^. Similarly, a study of data for children aged 12–16 years in the Early Childhood Longitudinal Study initiated in 2007 found increased odds of parent-reported mental health issues, emotional misconduct, peer problems and less optimal pro-social behaviour for any level of HFI compared with no HFI^(78)^. Another high-quality analysis of NHANES data for 12- to 17-year-olds in the USA more specifically explored the odds of anxiety when exposed to HFI and found that HFI was associated with greater odds of experiencing anxiety compared with no HFI^(77)^. A low-quality study explored depressive moods for children aged 10–18 years using the Korea National Health and Nutrition Examination Survey 2012–2019 dataset and found that for girls, there was a statistically significant association with any level of HFI^(58)^. Analyses of these large datasets consistently found associations between HFI and poor mental health outcomes.

One study explored the association between HFI and self-esteem and self-efficacy. A moderate-quality study using the Canadian Children’s Lifestyle and School Performance Study II dataset of 10- to 11-year-olds in Nova Scotia, Canada, found that in adjusted analyses, children with any level of HFI had greater odds of low self-esteem and self-efficacy to make healthy choices^(55)^. These findings were not present when the analysis was adjusted for covariates, including the child’s sex, body size, caregiver’s education level, household income and rurality^(55)^.

In terms of the use of mental health services, a high-quality study linking the Canadian Community Health Survey dataset to provincial health records found that the use of mental health or substance use service usage was associated with HFI for children aged 1–17 years; the strength of the association increased with the level of HFI severity^(30)^. Effect sizes were smaller but remained statistically significant when prevalence ratios were adjusted for covariates^(30)^.

#### Sleep

A small, high-quality cross-sectional study including American Indian adolescents through the Native American Youth Sleep Health and Wellness study^(69)^ found that adolescents with any level of HFI had more sleep disturbances than their food-secure peers^(69)^. Another small, high-quality cross-sectional study examined pre-school aged children in Headstart Preschools in Pennsylvania, USA, and found that preschoolers with any level of HFI had increased odds of poor sleep quality, but this association was not seen when the analysis was adjusted for covariates^(79)^.

#### Diet

In terms of overall diet quality, one large high-quality study in Canada found that children experiencing HFI had lower scores for diet quality^(56)^. No association between HFI and diet quality was found by three other studies using NHANES data in the USA^(74,80,82)^. Findings for daily energy intake were mixed, where two small high-quality studies found that daily energy intake was higher^(84)^ and lower^(82)^ for children experiencing HFI. Two other studies found no associations or differences between HFI and energy intake^(80,83)^.

Fruit and vegetable intake had the most marked difference for children experiencing HFI and their food-secure peers, but results were still mixed. While four studies (three high quality and one moderate quality) found that fruit and vegetable consumption was lower for children with HFI^(56,74,81,82)^, another three studies (two high quality and one moderate quality) found no difference^(80,83,84)^. Findings for protein and added sugar intake were also mixed. Two high-quality studies found that the proportion of energy intake from protein^(84)^ and protein from seafood and plant sources^(74)^ was lower for children with HFI, and a low-quality study also found lower protein intake for girls with HFI^(58)^. Two other studies, including one high and one moderate quality, did not find a difference in protein intake. Two smaller high-quality studies found differences in sugar intake for children experiencing HFI, specifically in terms of sugar sweetened beverages^(82)^ and added sugars^(84)^. However, no difference in sugar intake was observed by four other studies^(56,74,80,83)^. There were no observed differences between children experiencing HFI and their food secure peers for intake of whole grains^(74,80,83,84)^, Na^(56,74,80,83,84)^, fats^(56,58,74,80,83,84)^ or fibre^(56,58,83,84)^.

In terms of micronutrients, two studies reported findings for boys and girls separately^(58,80)^. A moderate quality study found that for children with HFI, boys had a lower intake of vitamin D, Zn, choline, and Mg, and girls had a lower Ca intake^(80)^. A low-quality study found that boys had lower vitamin A intake and girls had lower niacin intake when experiencing HFI^(58)^. A third moderate quality study found no difference in micronutrient intake for children experiencing HFI^(56)^.

#### Anaemia

Relevant to dietary outcomes, four studies reported on the association between HFI and anaemia or markers of Fe deficiency, with mixed findings^(51,57,80,85)^. Two analysed large population-level datasets, including a moderate-quality cross-sectional analysis of NHANES data for children aged 1–18 years in the USA^(80)^ and a high-quality cohort analysis of data for toddlers from a state food assistance program, the Special Supplemental Nutrition Program for Women, Infants, and Children in Massachusetts, USA^(85)^; odds of anaemia were increased in toddlers^(85)^, but this association was not seen across age groups^(80)^. Additionally, a small, high-quality cross-sectional study that assessed anaemia for children living in shelters while experiencing houselessness in Paris, France found an increased prevalence of anaemia with any level of HFI^(57)^. Finally, a small high-quality cross-sectional study of Inuit adolescents in Nunavik, Canada found no association between HFI and anaemia or Fe deficiency^(51)^.

## Discussion

Findings from this review support the hypothesis that exposure to HFI during childhood and/or adolescence is associated with a wide range of negative health outcomes, as evidenced by many high-quality studies. Although there is substantial heterogeneity in terms of HFI measurement and covariate adjustment across studies, findings indicate that HFI is related to negative health outcomes including general health status, asthma, dental caries and mental health. Findings for cardiometabolic health, diet and anaemia are mixed across studies. While challenging to establish causality through observational studies, findings align with many of the Bradford Hill criteria for causal pathways^(86)^. Particularly, findings are strong, consistent, plausible, show evidence of a clear gradient with increased levels of HFI or ‘dose-response’ and align with evidence for health effects associated with HFI for adults^(87)^. Additional high-quality longitudinal studies will provide clarity on the magnitude of these effects. The findings of this review support the importance of taking action to prevent and mitigate the negative health effects of HFI in childhood and adolescence.

In high-income countries, common interventions to address HFI include food banks or food pantries^(88)^. Given that many food banks or food pantries rely on donations and volunteer efforts, they are often unable to meet community needs in times of crises, and research evidence has failed to demonstrate that they reduce the prevalence of HFI^(88)^. On the other hand, policy interventions that aim to improve the financial circumstances for very low-income households have been shown to reduce HFI prevalence^(89,90)^. Effective policy interventions require integration across multiple sectors, including social, economic, health and agriculture, and across all levels of government^(90)^. In terms of reducing racial health inequities, a systematic review of structural interventions found that policies that increase income for low-income households had a clearer effect on health outcomes than nutrition programs that focused on access to food^(91)^.

The variation in the measurement of HFI added to the difficulty of comparing results across studies. Varying thresholds for defining food security *v*. insecurity may explain some heterogeneity in the findings across studies. In instances where a gradient of severity of the outcome of interest according to increasing levels of HFI was reported, findings varied in relation to health outcomes. In some cases, there was a clear gradient effect of increased risk with increasing severity of HFI^(52,76)^, but in others, the observed effect was not necessarily linear^(67)^. Further, there were instances when authors differentiated levels of HFI in the measurement, but findings were grouped and reported as a binary. Within in some of these studies, marginal food insecurity was grouped with food security, but the health impacts of experiencing marginal food insecurity can be significant, and researchers have emphasised the importance of differentiating it from food security^(20,92,93)^. To advance future evidence synthesis for health outcomes and HFI, there is a need for consistent measurement and analysis of HFI within the academic community in this field, particularly in terms of defining threshold categories for levels of HFI.

Covariate adjustment also varied widely among the included studies, adding to the heterogeneity across studies and challenging the synthesis of findings. While adjustment for confounding factors is critical in establishing the independent role of HFI in the health outcome, we emphasise caution against over-adjustment of analyses. When numerous inter-related socioeconomic factors are adjusted for the same analysis, there is potential to over adjust, resulting in attenuation of the true effect of HFI. This is especially true when factors are collinear or intermediaries on the causal pathway between HFI and health outcomes. Careful thought is needed to determine appropriate selection of confounders for adjustment. Differences in approaches to adjustment for confounding across studies may explain some of the heterogeneity in findings. Given the inclusion of studies from several different countries, other factors related to setting and context were also likely to influence study results and affect comparison between studies. Nevertheless, even with the wide variation in covariate adjustment in the studies included in this review, an increased risk of negative health outcomes after being exposed to HFI was demonstrated in most studies.

In literature related to HFI among children and adolescents, it is often cited that adults protect children in the household from food deprivation^(28,94–96)^. This may partly explain why findings are less clear for physical and dietary-related outcomes but clearer for mental health outcomes. Even if adults in food-insecure households are able to prevent younger members from experiencing food deprivation, children and youth in these households may be highly sensitive to and affected by stressors in the household which could compromise their mental health^(97)^.

It is important to note that the definition and measurement of HFI in the studies included is specific to income-related constraints. Authors acknowledge that this represents a Western and colonial worldview of HFI, data collection and reporting and is not inclusive of traditional Indigenous views and ways of knowing about food security and food procurement. This is an important consideration in the colonial public health context.

### Limitations of this review

There are several important limitations to this rapid systematic review. Heterogeneity in HFI measures and analysis posed a challenge for developing key messaging related to health outcomes. Additionally, using a rapid review approach may increase the risk of bias in the review findings. Modifications to the full systematic review approach include using a single screener to determine eligibility of retrieved studies, with error checking by artificial intelligence. Second reviewers were not blinded to data extraction and quality assessment completed by the first reviewer. The potential bias introduced by these modifications is likely minimal, given the efforts made to pilot a subset of references for screening and data extraction prior to completion by a single reviewer^(98)^.

### Conclusion

HFI continues to pose a major public health challenge, particularly among households with children and youth^(99–^ ^101)^. While there is wide variability in measurement, this rapid systematic review’s findings indicate important trends related to negative health outcomes associated with experiencing HFI during childhood and adolescence. This review provides evidence for the longer-term negative physical and mental health effects of HFI during this vulnerable life stage. Public health can use the findings of this review to influence policy-level actions by raising awareness about the importance of addressing rising rates of HFI and mitigating the harmful effects of HFI on children and adolescents.

## Supporting information

Clark et al. supplementary material Clark et al. supplementary material

## Supplementary material

For supplementary material accompanying this paper visit https://doi.org/10.1017/S1368980025101109.
