# Supplementary material for: Health outcomes following childhood or adolescent exposure to household food insecurity: a rapid systematic review
Source: Public Health Nutr. 2025 Sep 19;28(1):e165. doi: 10.1017/S1368980025101109 (PMC12722076; doi:10.1017/S1368980025101109)
Supplement: Clark et al. supplementary material [file S1368980025101109sup001.docx]

**Appendix A: Search Strategy**

On October 3, 2023, the following databases were searched using the search terms listed.

Database(s): Ovid MEDLINE(R) and Epub Ahead of Print, In-Process, In-Data-Review & Other Non-Indexed Citations, Daily and Versions 1946 to October 03, 2023

| # | Searches |
| --- | --- |
| 1 | food insecurity/ |
| 2 | ("want of food" or "lack of food" or "lacking food" or food scarcity or "food shortfall").ti. |
| 3 | (exp food/ or food ingredients/) and (barrier$ or hurdle$ or impediment$ or obstacle$).ti,kf. |
| 4 | (food$ adj3 (barrier$ or depriv* or desert* or hardship* or inaccess* or inequalit* or inequit* or insecur* or insufficien* or scarc* or lacking or "lack of" or poverty or poor or "no access*" or unavailab* or unaccess*)).ti,kf. |
| 5 | ((barrier$ or depriv* or hardship* or inaccess* or inequalit* or inequit* or insufficien* or scarc* or insecur* or lacking or "lack of" or poverty or poor or "no access" or unaccess* or unavail*) and ((healthy or nutritious or nutrition*) adj1 (diet$ or food$ or ingredient$))).ti,kw,kf. |
| 6 | (depriv* adj3 (food$ or grocer* or bread or dairy or grains or greens or fruit$ or meat$ or meal$ or milk or produce or vegetabl*)).ti,kw,kf. or (depriv* adj3 (food$ or grocer* or bread or dairy or grains or greens or fruit$ or meat$ or meal$ or milk or produce or vegetabl*)).ab. /freq=2 |
| 7 | ((barrier$ or hurdle$ or impediment$ or obstacle$) adj3 (food$ or grocer* or ((buy* or consum* or eat* or fresh or get* or obtain* or purchas*) adj1 (bread or dairy or grains or greens or fruit$ or meat$ or meal$ or milk or produce or vegetabl*)))).ti,kw,kf. or ((barrier$ or hurdle$ or impediment$ or obstacle$) adj3 (food$ or grocer* or ((buy* or consum* or eat* or fresh or get* or obtain* or purchas*) adj1 (bread or dairy or grains or greens or fruit$ or meat$ or meal$ or milk or produce or vegetabl*)))).ab. /freq=2 |
| 8 | or/1-7 [ ***1 - food insecurity SH and food or healthy food consumption barriers (KW)] |
| 9 | (food assistance/ or (food aid or food assistance or food stamp* or food voucher* or foodbank* or going hungry or food bank* or community pantry or nutrition* assist* or SNAP or soup kitchen*).ti,kw,kf.) not medline.st. [*** 2 - reliant on external supports for access to food] |
| 10 | (Child Poverty/ or "aid to families with dependent children"/ or relief work/ or Public Housing/ or Social Welfare/ or Poverty Areas/ or Poverty/ or ((poor adj3 (famil* or household* or parent* or mom or moms or mother* or dad or dads or father*)) or poverty or low income).ti.) and ((food or grocer* or bread or dairy or fruit or greens or meals or produce or vegetable*) adj3 (access* or economical or equalit* or equit* or secur* or sufficien* or suppl*)).ti,kf. |
| 11 | (Child Poverty/ or "aid to families with dependent children"/ or Poverty Areas/ or Poverty/ or relief work/ or Public Housing/ or Social Welfare/ or ((poor adj3 (famil* or household* or parent* or mom or moms or mother* or dad or dads or father*)) or poverty or low income).ti.) and (access to healthy foods/ or artificially sweetened beverages/ or caloric intake/ or carbonated beverages/ or candy/ or caloric intake/ or child nutritional physiological phenomena/ or Cooking/ or diet/ or "Diet, Food, and Nutrition"/ or dietary fats/ or Diet, Cariogenic/ or Diet, High-Fat/ or Diet, Atherogenic/ or diet, healthy/ or dietary fats/ or dietary intake/ or feeding behavior/ or exp Food/ or Food, Processed/ or fruit/ or Food Analysis/ or Food Chain/ or Food Deprivation/ or Food Safety/ or Food Ingredients/ or food preferences/ or Food Quality/ or food security/ or Food Services/ or food supply/ or hunger/ or nutritional physiological phenomena/ or nutritional requirements/ or nutritional status/ or nutritive value/ or nutrition disorders/ or overnutrition/ or overweight/ or recommended dietary allowances/ or salads/ or Snacks/ or sugar-sweetened beverages/ or vegetables/ or ((diet* or food$ or eat* or fruit$ or meal$ or produce$ or vegetable$) adj3 (choice$ or choos* or habit$ or option$ or prefer*)).ti,kf. or (poor diet* or ((healthy or nutritious or nutrition* or unhealthy or non-nutritious) adj1 (diet$ or food$ or ingredient$ or meal$))).ti,kw,kf,hw.) |
| 12 | ((low income or poverty or (poor adj1 (dad or dads or fathers or famil* or mother or mom or moms or parent*))) adj3 (bread$ or (consum* adj2 produce) or cooking or cookery or dairy or diet$ or eat* or food$ or fruit or fruits or grain$ or greens or ingredient$ or legume* or meal$ or meat or nutrition* or overnutrition or undernutrition or vegetable*)).ab. /freq=2 |
| 13 | (nutrition* adj1 (at risk or insecur*)).ti,kf. |
| 14 | or/10-13 [*** 3 - low income/poverty and food/diet or nutritionally at risk] |
| 15 | ((grocer* or bread$ or dairy or food$ or fruit$ or greens or meal$ or meat$ or produce or vegetable*) adj3 (affordability or cost prohobitive or markup$ or mark-up or price goug* or "too expensive" or "too costly" or unaffordab* or (high* adj1 (cost$ or expens* or pric*)))).ti,kf. [*** -4 cost of food as a barrier] |
| 16 | food deserts/ or (((inaccess* or unaccess*) adj1 food) or food desert*).ti,kw,kf. |
| 17 | (((barrier$ or depriv* or desert or hurdle$ or impediment$ or inaccess* or lacking or "lack of" or obstacle$ or unaccess* or unavail*) adj3 (grocer* or supermarket$)) or ((barrier$ or depriv* or desert or hurdle$ or impediment$ or inaccess* or lacking or "lack of" or obstacle$ or unaccess* or unavail*) adj3 ((food or grocer*) adj1 (chain* or market or retail* or shop* or store* or suppl* or vendor$)))).ti,ab. |
| 18 | or/16-17 [***5 - food desert, lack of access to grocery stores] |
| 19 | Geography/ or Geographic Information Systems/ or geographic mapping/ or spatial analysis/ or residence characteristics/ or built environment/ or home environment/ or Poverty Areas/ or area$.ti. or (neighbourhood or neighborhood).ti,kw,kf. |
| 20 | ("built environment" or foodscape or "food environment$" or "nutritional environment$" or "distance to" or "distance from" or near or nearby or "close by" or "close to" or "far from" or "far away" or "walking distance" or "travel time" or (transportation adj2 (access* or route$)) or proximity).ti,kw,kf. |
| 21 | supermarkets/ or food services/ or fast foods/ or restaurants/ or Food Dispensers, Automatic/ |
| 22 | (((convenienc* or corner or grocer*) adj1 store*) or grocer* or restaurant* or supermarket$ or fast food$ or ((food* or grocer*) adj3 (chain$ or convenien* or depot* or distribut* or market$ or merchant$ or outlet* or retail* or sale$ or seller$ or shop* or store$ or suppl* or trade* or vendor$)) or nutrition* environment).ti,kf,kw. |
| 23 | (or/19-20) and (or/21-22) [*** 6 - food geography or distance to stores] |
| 24 | (((food or nutrition*) adj1 (desert$ or swamp$)) or Obesogenic environment$).ti,kf,kw. or (((food or nutrition*) adj1 (desert$ or swamp$)) or Obesogenic environment$).ab. /freq=2 [*** 7 - poor food environment, food dump] |
| 25 | ((artificial or convenience or fast or healthy or high calorie or high fat or junk or low calorie or nutritious or option$ or pre-made or pre-packaged or processed or snack or sugar laden or sugar sweeten* or unhealthy) adj1 (beverag* or food$)).ti,kf. |
| 26 | access to healthy foods/ or artificially sweetened beverages/ or caloric intake/ or carbonated beverages/ or candy/ or caloric intake/ or child nutritional physiological phenomena/ or Cooking/ or diet/ or "Diet, Food, and Nutrition"/ or dietary fats/ or Diet, Cariogenic/ or Diet, High-Fat/ or Diet, Atherogenic/ or diet, healthy/ or dietary fats/ or dietary intake/ or feeding behavior/ or exp Food/ or Food, Processed/ or fruit/ or Food Analysis/ or Food Chain/ or Food Deprivation/ or Food Safety/ or Food Ingredients/ or food preferences/ or Food Quality/ or food security/ or Food Services/ or food supply/ or hunger/ or nutritional physiological phenomena/ or nutritional requirements/ or nutritional status/ or nutritive value/ or nutrition disorders/ or overnutrition/ or overweight/ or recommended dietary allowances/ or salads/ or Snacks/ or sugar-sweetened beverages/ or vegetables/ or ((diet* or food$ or eat* or fruit$ or meal$ or produce$ or vegetable$) adj3 (choice$ or choos* or habit$ or option$ or prefer*)).ti,kf. or (poor diet* or ((healthy or nutritious or nutrition* or unhealthy or non-nutritious) adj1 (diet$ or food$ or ingredient$ or meal$))).ti,kw,kf,hw. |
| 27 | ((lacking or "lack of" or "no" or proximity or without) adj3 (bakery or bread or butcher shop or deli or grocer* or food retail* or food seller* or food store* or food vendor$ or fruit$ or health$ food$ or nutritious food$ or produce or supermarket$ or vegetable$)).ti,kw,kf. or ((lacking or "lack of" or "no" or without) adj3 (bakery or bread or butcher shop or deli or grocer* or food retail* or food seller* or food store* or food vendor$ or fruit$ or health$ food$ or nutritious food$ or produce or supermarket$ or vegetable$)).ab. /freq=2 |
| 28 | 19 and (25 or 26 or 27) [***8 - geography and food options, choices, diet etc - option 2] |
| 29 | access to healthy foods/ or diet, healthy/ or Diet, Fat-Restricted/ or nutritional requirements/ or nutritional status/ or recommended dietary allowances/ or balanced diet.ti,kf. or ((Cooking/ or diet/ or "Diet, Food, and Nutrition"/ or dietary intake/ or feeding behavior/) and (Dietary Fiber/ or food quality/ or functional food/ or food preference/ or fruit/ or nutritive value/ or salads/ or vegetables/ or whole grains/ or (fruit$ or high fiber or green$ or lean* meat$ or low* fat or low* sodium or produce or salad$ or vegetable$ or whole grain$).ti,kf.)) |
| 30 | (diet* or food prefer*).ti,kf,hw. and ((high adj2 fiber) or ((low* or reduce*) adj2 (fat$ or salt or sodium or sugar$))).ti,kf. |
| 31 | (Nutrition Policy/ or (food pyramid or ((diet* or eating or food or nutrition*) adj2 (guide* or reccomend*))).ti,kf.) and (Patient Compliance/ or (adher* or comply*).ti,kf.) |
| 32 | or/29-31 [nutritious food] |
| 33 | (barrier$ or hurdle$ or impediment$ or obstacle$).ti. |
| 34 | *Transportation/ or Transportation Facilities/ or ((insecur* or lacking or "lack of") adj3 (automobile$ or car$ or bus* or travel* or transport* or vechicle$)).ti,kw,kf. |
| 35 | economics, behavioral/ or poverty/ or risk factors/ or *socioeconomic factors/ or social determinants of health/ or (affordability or cost prohobit* or expens* or markup$ or mark-up or price* or pricing or price goug* or "too expensive" or "too costly" or unaffordab* or (high* adj1 (cost$ or expens* or pric*)) or ((diet* or food or grocer* or meal$ or produce or vegetable$) adj2 cost$)).ti. |
| 36 | Literacy/ or Health Literacy/ or Health Knowledge, Attitudes, Practice/ or self efficacy/ or ((cooking or diet* or food or health or nutrition*) adj2 (illit* or litera or knowledge or skill)).ti,kf,kw. [barriers] |
| 37 | 33 or 34 or 35 or 36 [generic barriers or identified factors influencing access to nutritious food ] |
| 38 | 32 and 37 [*** 9 - barriers to nutritious food] |
| 39 | ((automobile* or bus or car or fare or taxi or transit or transport*) adj3 (grocer* or food)).ti,kw,kf,hw. or ((automobile* or bus or car or fare or taxi or transit or transport*) adj3 (grocer* or food)).ab. /freq=2 |
| 40 | ((commut* or distance$ or distant or far or far away or time or travel* or barrier$ or hurdle$ or impediment* or mobility or obstacle$) adj3 (grocer* or food or supermarket$)).ti,kf,kw,ab. |
| 41 | ((commut* or distance$ or distant or far or far away or time or travel* or barrier$ or hurdle$ or impediment* or mobility or obstacle$) adj3 (food adj2 (market$ or retail* or shop* or store* or suppl*))).ti,kf,kw,ab. |
| 42 | ((commut* or distance$ or distant or far or far away or time or travel* or barrier$ or hurdle$ or impediment* or mobility or obstacle$) adj3 ((convenienc* or corner*) adj1 store*)).ti,kw,kf,ab. |
| 43 | ((commut* or distance$ or distant or far or far away or travel* or barrier$ or hurdle$ or impediment* or mobility or obstacle$) adj5 (bread or butcher$ or dairy or deli or delicatessen or grocer* or food or fruit$ or milk or produce or vegetable$)).mp. |
| 44 | ((secur* or insecur*) adj3 (travel* or transport*)).ti,kw,kf. and (diet, healthy/ or exp food/ or food supply/ or access to healthy foods/ or (grocer* or food).ti,kf.) |
| 45 | or/39-44 [KW - transportation barriers to food, grocery stores or healthy diet] |
| 46 | 8 or 9 or 14 or 15 or 18 or 23 or 24 or 28 or 38 or 45 |
| 47 | limit 46 to ("all child (0 to 18 years)" or "preschool child (2 to 5 years)" or "child (6 to 12 years)" or "adolescent (13 to 18 years)" or "young adult (19 to 24 years)") |
| 48 | adolescent/ or child/ or child, preschool/ or minors/ |
| 49 | adolescent health/ or child health/ |
| 50 | pediatrics/ or pediatric emergency medicine/ |
| 51 | (adolescent* or ((before or prior to preceding) adj3 (adulthood or maturity)) or child or children or early years or (early adj2 li?e$) or emerging adult$ or highschooler* or highschool age* or high school age* or preschooler* or pre-schooler$ or pediatric* or school age* or teen$ or teen-age* or teenage* or toddler$ or young adult*).ti,kw,kf,hw. |
| 52 | (("2" or "3" or "4" or "5" or "6" or "7" or "8" or "9" or "10" or "11" or "12" or "13" or "14" or "15" or "16" or "17" or "18" or "19" or "20" or "21") adj2 (age$ or year$ old$)).ti,kw,kf. |
| 53 | or/48-52 |
| 54 | 47 or (46 and 53) |
| 55 | (exp Africa/ or exp Caribbean Region/ or exp Central America/ or exp Latin America/ or exp South America/ or exp Asia/ or Developing Countries/) not (exp North America/ or exp Australia/ or exp Europe/ or exp Developed Countries/) |
| 56 | 54 not 55 |
| 57 | (((affluen* or developed or high income or wealthy or "well off" or western or G20 or australasia* or europ* or North Americ*) adj1 (countr* or nation$ or provinc* or state)) or ((developed or first or western) adj1 world)).ti,kw,kf,hw. |
| 58 | 54 and 57 |
| 59 | 56 or 58 |
| 60 | limit 59 to english language |
| 61 | limit 60 to yr="2013 -Current" |
| 62 | limit 61 to yr="2013 - 2015" |
| 63 | limit 61 to yr="2016 - 2018" |
| 64 | limit 61 to yr="2019 - 2020" |
| 65 | limit 61 to yr="2021 - 2022" |
| 66 | limit 61 to yr="2023 - 2023" |
| 67 | 62 or 63 or 64 or 65 or 66 |

Database(s): Embase 1980 to 2023 Week 39

| # | Searches |
| --- | --- |
| 1 | food insecurity/ or food shortage/ |
| 2 | ("want of food" or "lack of food" or "lacking food" or food scarcity or "food shortfall").ti. |
| 3 | (food access/ or food ingredient/ or exp food/) and (barrier$ or hurdle$ or impediment$ or obstacle$).ti,kf. |
| 4 | (food$ adj3 (barrier$ or depriv* or desert* or hardship* or inaccess* or inequalit* or inequit* or insecur* or insufficien* or scarc* or lacking or "lack of" or poverty or poor or "no access" or unavailab* or unaccess*)).ti,kf. |
| 5 | ((barrier$ or depriv* or hardship* or inaccess* or inequalit* or inequit* or insufficien* or scarc* or insecur* or lacking or "lack of" or poverty or poor or "no access" or unaccess* or unavail*) and ((healthy or nutritious or nutrition*) adj1 (diet$ or food$ or ingredient$))).ti,kw,kf. |
| 6 | (depriv* adj3 (food$ or grocer* or bread or dairy or grains or greens or fruit$ or meat$ or meal$ or milk or produce or vegetabl*)).ti,kw,kf. or (depriv* adj3 (food$ or grocer* or bread or dairy or grains or greens or fruit$ or meat$ or meal$ or milk or produce or vegetabl*)).ab. /freq=2 |
| 7 | ((barrier$ or hurdle$ or impediment$ or obstacle$) adj3 (food$ or grocer* or ((buy* or consum* or eat* or fresh or get* or obtain* or purchas*) adj1 (bread or dairy or grains or greens or fruit$ or meat$ or meal$ or milk or produce or vegetabl*)))).ti,kw,kf. or ((barrier$ or hurdle$ or impediment$ or obstacle$) adj3 (food$ or grocer* or ((buy* or consum* or eat* or fresh or get* or obtain* or purchas*) adj1 (bread or dairy or grains or greens or fruit$ or meat$ or meal$ or milk or produce or vegetabl*)))).ab. /freq=2 |
| 8 | or/1-7 [ ***1 - food insecurity Subject headings or barriers to food, healthy food (KW)] |
| 9 | food assistance/ or (food aid or food assistance or food stamp* or food voucher* or foodbank* or going hungry or food bank* or community pantry or nutrition* assist* or SNAP or soup kitchen*).ti,kw,kf. [*** 2 - reliant on external supports for access to food] |
| 10 | (poverty/ or child poverty/ or extreme poverty/ or *housing/ or *social welfare/ or (public housing or welfare or (poor adj3 (famil* or household* or parent* or mom or moms or mother* or dad or dads or father*)) or poverty or low income).ti.) and ((food or grocer* or bread or dairy or fruit or greens or meals or produce or vegetable*) adj3 (access* or economical or equalit* or equit* or secur* or sufficien* or suppl*)).ti,kf. |
| 11 | (poverty/ or child poverty/ or extreme poverty/ or *housing/ or *social welfare/ or (public housing or welfare or (poor adj3 (famil* or household* or parent* or mom or moms or mother* or dad or dads or father*)) or poverty or low income).ti.) and (healthy food access/ or food access/ or artificially sweetened beverage/ or caloric intake/ or dietary intake/ or carbonated beverage/ or candy/ or child nutrition/ or nutrition/ or exp cooking/ or food processing/ or exp diet/ or high-glucose diet/ or "high fat/high fructose diet"/ or diet composition/ or high salt diet/ or high calorie diet/ or diet-induced obesity/ or "high fat/high sucrose diet"/ or high-fructose diet/ or healthy diet/ or fat intake/ or atherogenic diet/ or lipid diet/ or feeding behavior/ or exp food/ or food handling/ or processed food/ or food analysis/ or fruit consumption/ or vegetable consumption/ or food chain/ or food deprivation/ or dietary pattern/ or food safety/ or food ingredient/ or food preference/ or food quality/ or catering service/ or home delivered meal/ or restaurant/ or "takeaway (food)"/ or hunger/ or nutritional requirement/ or food guide pyramid/ or nutrition/ or adolescent nutrition/ or child nutrition/ or meal/ or nutrient availability/ or nutrient content/ or nutrient supply/ or nutrient uptake/ or nutritional assessment/ or nutritional value/ or overnutrition/ or nutritional disorder/ or obesity/ or salad/ or fast food/ or sugar-sweetened beverage/ or vegetable/) |
| 12 | (poverty/ or child poverty/ or extreme poverty/ or *housing/ or *social welfare/ or (public housing or welfare or (poor adj3 (famil* or household* or parent* or mom or moms or mother* or dad or dads or father*)) or poverty or low income).ti.) and ((diet* or food$ or eat* or fruit$ or meal$ or produce$ or vegetable$) adj3 (choice$ or choos* or habit$ or option$ or prefer*)).ti,kf. |
| 13 | (poverty/ or child poverty/ or extreme poverty/ or *housing/ or *social welfare/ or (public housing or welfare or (poor adj3 (famil* or household* or parent* or mom or moms or mother* or dad or dads or father*)) or poverty or low income).ti.) and (poor diet* or ((healthy or nutritious or nutrition* or unhealthy or non-nutritious) adj1 (diet$ or food$ or ingredient$ or meal$))).ti,kw,kf,hw. |
| 14 | 10 or 11 or 12 or 13 |
| 15 | ((low income or poverty or (poor adj1 (dad or dads or fathers or famil* or mother or mom or moms or parent*))) adj3 (bread$ or (consum* adj2 produce) or cooking or cookery or dairy or diet$ or eat* or food$ or fruit or fruits or grain$ or greens or ingredient$ or legume* or meal$ or meat or nutrition* or overnutrition or undernutrition or vegetable*)).ab. /freq=2 |
| 16 | (nutrition* adj1 (at risk or insecur*)).ti,kf. |
| 17 | 14 or 15 or 16 [low income/poverty and food/diet OR nutritionally at risk] |
| 18 | ((grocer* or bread$ or dairy or food$ or fruit$ or greens or meal$ or meat$ or produce or vegetable*) adj3 (affordability or cost prohibitive or markup$ or mark-up or price goug* or "too expensive" or "too costly" or unaffordab* or (high* adj1 (cost$ or expens* or pric*)))).ti,kf. [*** -4 cost of food as a barrier] |
| 19 | food desert/ or (((inaccess* or unaccess*) adj1 food) or food desert*).ti,kw,kf. |
| 20 | (((barrier$ or depriv* or desert or hurdle$ or impediment$ or inaccess* or lacking or "lack of" or obstacle$ or unaccess* or unavail*) adj3 (grocer* or supermarket$)) or ((barrier$ or depriv* or desert or hurdle$ or impediment$ or inaccess* or lacking or "lack of" or obstacle$ or unaccess* or unavail*) adj3 ((food or grocer*) adj1 (chain* or market or retail* or shop* or store* or suppl* or vendor$)))).ti,ab. |
| 21 | or/19-20 [- food desert, lack of access to grocery stores] |
| 22 | geographic distribution/ or geography/ or geographic information system/ or geographic mapping/ or spatial analysis/ or residence characteristics/ or built environment/ or home environment/ or neighborhood/ or neighborhood characteristic/ or (area$ or neighbourhood or neighborhood).ti. |
| 23 | ("built environment" or foodscape or "food environment$" or "nutritional environment$" or "distance to" or "distance from" or near or nearby or "close by" or "close to" or "far from" or "far away" or "walking distance" or "travel time" or (transportation adj2 (access* or route$)) or proximity).ti,kw,kf. |
| 24 | supermarket/ or catering service/ or fast food/ or restaurant/ or food dispenser/ |
| 25 | (((convenienc* or corner or grocer*) adj1 store*) or grocer* or restaurant* or supermarket$ or fast food$ or ((food* or grocer*) adj3 (chain$ or convenien* or depot* or distribut* or market$ or merchant$ or outlet* or retail* or sale$ or seller$ or shop* or store$ or suppl* or trade* or vendor$)) or nutrition* environment).ti,kf,kw. |
| 26 | (or/22-23) and (or/24-25) |
| 27 | (((food or nutrition*) adj1 (desert$ or swamp$)) or Obesogenic environment$).ti,kf,kw. or (((food or nutrition*) adj1 (desert$ or swamp$)) or Obesogenic environment$).ab. /freq=2 [*** 7 - poor food environment, food dump] |
| 28 | ((artificial or convenience or fast or healthy or high calorie or high fat or junk or low calorie or nutritious or option$ or pre-made or pre-packaged or processed or snack or sugar laden or sugar sweeten* or unhealthy) adj1 (beverag* or food$)).ti,kf. |
| 29 | healthy food access/ or food access/ or artificially sweetened beverage/ or caloric intake/ or dietary intake/ or carbonated beverage/ or candy/ or child nutrition/ or nutrition/ or exp cooking/ or food processing/ or diet/ or high-glucose diet/ or "high fat/high fructose diet"/ or diet composition/ or high salt diet/ or high calorie diet/ or diet-induced obesity/ or "high fat/high sucrose diet"/ or high-fructose diet/ or healthy diet/ or fat intake/ or atherogenic diet/ or lipid diet/ or feeding behavior/ or exp food/ or food handling/ or processed food/ or food analysis/ or fruit consumption/ or vegetable consumption/ or food chain/ or food deprivation/ or dietary pattern/ or food safety/ or food ingredient/ or food preference/ or food quality/ or catering service/ or home delivered meal/ or restaurant/ or "takeaway (food)"/ or hunger/ or nutritional requirement/ or food guide pyramid/ or nutrition/ or adolescent nutrition/ or child nutrition/ or meal/ or nutrient availability/ or nutrient content/ or nutrient supply/ or nutrient uptake/ or nutritional assessment/ or nutritional value/ or overnutrition/ or nutritional disorder/ or obesity/ or salad/ or fast food/ or sugar-sweetened beverage/ or vegetable/ |
| 30 | ((diet* or food$ or eat* or fruit$ or meal$ or produce$ or vegetable$) adj3 (choice$ or choos* or habit$ or option$ or prefer*)).ti,kf. or (poor diet* or ((healthy or nutritious or nutrition* or unhealthy or non-nutritious) adj1 (diet$ or food$ or ingredient$ or meal$))).ti,kw,kf,hw. |
| 31 | ((lacking or "lack of" or "no" or proximity or without) adj3 (bakery or bread or butcher shop or deli or grocer* or food retail* or food seller* or food store* or food vendor$ or fruit$ or health$ food$ or nutritious food$ or produce or supermarket$ or vegetable$)).ti,kw,kf. or ((lacking or "lack of" or "no" or without) adj3 (bakery or bread or butcher shop or deli or grocer* or food retail* or food seller* or food store* or food vendor$ or fruit$ or health$ food$ or nutritious food$ or produce or supermarket$ or vegetable$)).ab. /freq=2 |
| 32 | 22 and (or/28-31) |
| 33 | healthy food access/ or healthy diet/ or nutritional requirement/ or dietary reference intake/ or low fat diet/ or (((health or nutritious) adj1 food$) or balanced diet).ti,kf. |
| 34 | (exp cooking/ or diet/ or diet composition/ or dietary intake/ or feeding behavior/) and (dietary fiber/ or food quality/ or exp fruit/ or nutrition/ or nutrient supply/ or nutrient uptake/ or nutritional health/ or nutritional requirement/ or nutritional status/ or exp vegetable/ or salad/ or nutritional value/ or whole grain/ or (fruit$ or high fiber or green$ or lean* meat$ or low* fat or low* sodium or produce or salad$ or vegetable$ or whole grain$).ti,kf.) |
| 35 | (diet* or food prefer*).ti,kf,hw. and ((high adj2 fiber) or ((low* or reduce*) adj2 (fat$ or salt or sodium or sugar$))).ti,kf. |
| 36 | (nutrition policy/ or food guide pyramid/ or (food pyramid or ((diet* or eating or food or nutrition*) adj2 (guide* or reccomend*))).ti,kf.) and (protocol compliance/ or patient compliance/ or (adher* or comply*).ti,kf.) |
| 37 | 33 or 34 or 35 or 36 |
| 38 | (barrier$ or hurdle$ or impediment$ or obstacle$).ti. |
| 39 | "traffic and transport"/ or ((insecur* or lacking or "lack of") adj3 (automobile$ or car$ or bus* or travel* or transport* or vechicle$)).ti,kw,kf. |
| 40 | behavioral economics/ or extreme poverty/ or child poverty/ or poverty level/ or poverty/ or risk factor/ or socioeconomics/ or "social determinants of health"/ or (affordab* or cost prohibit* or expens* or markup$ or mark-up$ or pricing or price or price goug* or too expensive or too costly or unaffordab* or (high* adj1 (cost$ or expens* or pric*)) or ((diet or food or grocer* or produce or vegetable$) adj2 cost$)).ti. |
| 41 | health literacy/ or literacy/ or (((cooking or food or nutrition) adj1 (literacy or skill$)) or self efficacy).ti,kw. |
| 42 | or/38-41 [generic barriers or identified factures influencing access to nutritious food] |
| 43 | 37 and 42 [barriers to nutritious food, healthy diet] |
| 44 | ((automobile* or bus or car or fare or taxi or transit or transport*) adj3 (grocer* or food)).ti,kw,kf,hw. or ((automobile* or bus or car or fare or taxi or transit or transport*) adj3 (grocer* or food)).ab. /freq=2 |
| 45 | ((commut* or distance$ or distant or far or far away or time or travel* or barrier$ or hurdle$ or impediment* or mobility or obstacle$) adj3 (grocer* or food or supermarket$)).ti,kf,kw,ab. |
| 46 | ((commut* or distance$ or distant or far or far away or time or travel* or barrier$ or hurdle$ or impediment* or mobility or obstacle$) adj3 (food adj2 (market$ or retail* or shop* or store* or suppl*))).ti,kf,kw,ab. |
| 47 | ((commut* or distance$ or distant or far or far away or time or travel* or barrier$ or hurdle$ or impediment* or mobility or obstacle$) adj3 ((convenienc* or corner*) adj1 store*)).ti,kw,kf,ab. |
| 48 | ((commut* or distance$ or distant or far or far away or travel* or barrier$ or hurdle$ or impediment* or mobility or obstacle$) adj5 (bread or butcher$ or dairy or deli or delicatessen or grocer* or food or fruit$ or milk or produce or vegetable$)).mp. |
| 49 | ((secur* or insecur*) adj3 (travel* or transport*)).ti,kw,kf. and (diet, healthy/ or exp food/ or food supply/ or access to healthy foods/ or (grocer* or food).ti,kf.) |
| 50 | or/44-49 [barriers to a healthy diet and other barriers with kw and ab] |
| 51 | 8 or 9 or 10 or 14 or 17 or 21 or 26 or 32 or 50 |
| 52 | limit 51 to (child or preschool child <1 to 6 years> or school child <7 to 12 years> or adolescent <13 to 17 years>) |
| 53 | adolescent/ or child/ or preschool child/ or "minor (person)"/ |
| 54 | adolescent health/ or child health/ |
| 55 | pediatric emergency medicine/ or pediatrics/ |
| 56 | (adolescent* or ((before or prior to preceding) adj3 (adulthood or maturity)) or child or children or early years or (early adj2 li?e$) or emerging adult$ or highschooler* or highschool age* or high school age* or preschooler* or pre-schooler$ or pediatric* or school age* or teen$ or teen-age* or teenage* or toddler$ or young adult*).ti,kw,kf,hw. |
| 57 | (adolescent* or ((before or prior to preceding) adj3 (adulthood or maturity)) or child or children or early years or (early adj2 li?e$) or emerging adult$ or highschooler* or highschool age* or high school age* or preschooler* or pre-schooler$ or pediatric* or school age* or teen$ or teen-age* or teenage* or toddler$ or young adult*).ti,kw,kf,hw. |
| 58 | (("2" or "3" or "4" or "5" or "6" or "7" or "8" or "9" or "10" or "11" or "12" or "13" or "14" or "15" or "16" or "17" or "18" or "19" or "20" or "21") adj2 (age$ or year$ old$)).ti,kw,kf. |
| 59 | young adult/ |
| 60 | 52 or (51 and (or/53-59)) |
| 61 | (exp Africa/ or exp South America/ or exp "South and Central America"/ or exp Asia/ or exp Caribbean Islands/ or developing country/) not (exp North America/ or exp Europe/ or exp "Australia and New Zealand"/ or exp Australia/ or developed country/) |
| 62 | 60 not 61 |
| 63 | (((affluen* or developed or high income or wealthy or "well off" or western or G20 or australasia* or europ* or North Americ*) adj1 (countr* or nation$ or provinc* or state)) or ((developed or first or western) adj1 world)).ti,kw,kf,hw. |
| 64 | 60 and 63 |
| 65 | 62 or 64 |
| 66 | limit 65 to english language |
| 67 | limit 66 to yr="2013 -Current" |
| 68 | limit 67 to yr="2013 - 2015" |
| 69 | limit 67 to yr="2016 - 2017" |
| 70 | limit 67 to yr="2018 - 2019" |
| 71 | limit 67 to yr="2020 - 2021" |
| 72 | limit 67 to yr="2022 - 2022" |
| 73 | limit 67 to yr="2023 - 2023" |
| 74 | 68 or 69 or 70 or 71 or 72 or 73 |

Database(s): APA PsycInfo 1987 to September Week 4 2023

| # | Searches |
| --- | --- |
| 1 | food insecurity/ |
| 2 | ("want of food" or "lack of food" or "lacking food" or food scarcity or "food shortfall").ti. |
| 3 | exp Food/ and (barrier$ or hurdle$ or impediment$ or obstacle$).ti,id. |
| 4 | (food$ adj3 (barrier$ or depriv* or desert* or hardship* or inaccess* or inequalit* or inequit* or insecur* or insufficien* or scarc* or lacking or "lack of" or poverty or poor or unaccess*)).ti,id. |
| 5 | ((barrier$ or depriv* or expens* or hardship* or inaccess* or inequalit* or inequit* or insufficien* or scarc* or insecur* or lacking or "lack of" or markup$ or mark-up or price goug* or poverty or poor or sufficien$ or suppl* or unafford* or unavail*) and ((healthy or nutritious or nutrition*) adj1 (diet$ or food$ or ingredient$))).ti,id. |
| 6 | (depriv* adj3 (food$ or grocer* or bread or dairy or grains or greens or fruit$ or meat$ or meal$ or milk or produce or vegetabl*)).ti,id. or (depriv* adj3 (food$ or grocer* or bread or dairy or grains or greens or fruit$ or meat$ or meal$ or milk or produce or vegetabl*)).ab. /freq=2 |
| 7 | ((barrier$ or hurdle$ or impediment$ or obstacle$) adj3 (food$ or grocer* or ((buy* or consum* or eat* or fresh or get* or obtain* or purchas*) adj1 (bread or dairy or grains or greens or fruit$ or meat$ or meal$ or milk or produce or vegetabl*)))).ti,id. or ((barrier$ or hurdle$ or impediment$ or obstacle$) adj3 (food$ or grocer* or ((buy* or consum* or eat* or fresh or get* or obtain* or purchas*) adj1 (bread or dairy or grains or greens or fruit$ or meat$ or meal$ or milk or produce or vegetabl*)))).ab. /freq=2 |
| 8 | or/1-7 [1 - defining food insecurity - generic terms for lack of or barriers] |
| 9 | (food aid or food assistance or food stamp* or food voucher* or foodbank* or going hungry or food bank* or community pantry or nutrition* assist* or SNAP or soup kitchen*).ti,id. [2 - defining food insecurity by reliance on food assistance] |
| 10 | (poverty/ or poverty areas/ or ghettoes/ or economic disadvantage/ or lower income level/ or community welfare services/ or "welfare services (government)"/ or ((poor adj3 (famil* or household* or parent* or mom or moms or mother* or dad or dads or father*)) or poverty or low income).ti.) and ((food or grocer* or bread or dairy or fruit or greens or meals or produce or vegetable*) adj3 (access* or economical or equalit* or equit* or secur* or sufficien* or suppl*)).ti,id. |
| 11 | (poverty/ or poverty areas/ or ghettoes/ or economic disadvantage/ or lower income level/ or community welfare services/ or "welfare services (government)"/ or ((poor adj3 (famil* or household* or parent* or mom or moms or mother* or dad or dads or father*)) or poverty or low income).ti.) and ("beverages (nonalcoholic)"/ or binge eating/ or calories/ or Diets/ or drinking behavior/ or exp Eating Behavior/ or eating attitudes/ or food deprivation/ or fast food/ or exp Food/ or Food Intake/ or food preferences/ or food preparation/ or food safety/ or hunger/ or meat consumption/ or exp Nutrition/ or (exp Supply Chains/ and exp Food/) or (cooking or candy or artificially sweetened beverages or soft drink* or ((consum* or diet* or eat* or intake) adj2 (fat or high-fat)) or fruit$ or fast food$ or nutrition or overnutrition or salad or snacks or snacking or vegetable$).ti.) |
| 12 | ((low income or poverty or (poor adj1 (dad or dads or fathers or famil* or mother or mom or moms or parent*))) adj3 (bread$ or (consum* adj2 produce) or cooking or cookery or dairy or diet$ or eat* or food$ or fruit or fruits or grain$ or greens or ingredient$ or legume* or meal$ or meat or nutrition* or overnutrition or undernutrition or vegetable*)).ab. /freq=2 |
| 13 | (nutrition* adj1 (at risk or insecur*)).ti,id. |
| 14 | or/10-13 [defining food insecurity with poverty+ food/diet or nutritionally at risk] |
| 15 | ((grocer* or bread$ or dairy or food$ or fruit$ or greens or meal$ or meat$ or produce or vegetable*) adj3 (affordability or markup$ or mark-up or price goug* or "too expensive" or "too costly" or unaffordab* or (high* adj1 (cost$ or expens* or pric*)))).ti,id. [*** -4 cost of food as a barrier] |
| 16 | food deserts.mh. or (((inaccess* or unaccess*) adj1 food) or food desert*).ti,id. |
| 17 | (((access* or barrier$ or depriv* or desert or hurdle$ or impediment$ or inaccess* or lacking or "lack of" or obstacle$ or unaccess* or unavail*) adj3 (grocer* or supermarket$)) or ((access* or barrier$ or depriv* or desert or hurdle$ or impediment$ or inaccess* or lacking or "lack of" or obstacle$ or unaccess* or unavail*) adj3 ((food or grocer*) adj1 (chain* or market or retail* or shop* or store* or suppl* or vendor$)))).ti,ab. |
| 18 | or/16-17 [food desert, lack of access to grocery stores] |
| 19 | geography/ or exp Environmental Effects/ or exp neighborhoods/ or ghettoes/ or built environment/ or Home Environment/ or poverty areas/ or area$.ti. or (neighbourhood or neighborhood).ti,id. |
| 20 | ("built environment" or foodscape or "food environment$" or "nutritional environment$" or "distance to" or "distance from" or near or nearby or "close by" or "close to" or "far from" or "far away" or "walking distance" or "travel time" or (transportation adj2 (access* or route$)) or proximity).ti,id. |
| 21 | exp Fast Food/ or (exp food/ and (Business/ or retailing/ or exp Shopping/ or shopping centers/)) |
| 22 | (((convenienc* or corner or grocer*) adj1 store*) or grocer* or restaurant* or supermarket$ or fast food$ or ((food* or grocer*) adj3 (chain$ or convenien* or depot* or distribut* or market$ or merchant$ or outlet* or retail* or sale$ or seller$ or shop* or store$ or suppl* or trade* or vendor$)) or nutrition* environment).ti,id. |
| 23 | supermarket*.ti,id. |
| 24 | (or/19-20) and (or/21-23) [food geography or distance to food] |
| 25 | (((food or nutrition*) adj1 (desert$ or swamp$)) or Obesogenic environment$).ti,id. or (((food or nutrition*) adj1 (desert$ or swamp$)) or Obesogenic environment$).ab. /freq=2 [*** 7- poor food environment, food dump] |
| 26 | ((artificial or convenience or fast or healthy or high calorie or high fat or junk or low calorie or nutritious or option$ or pre-made or pre-packaged or processed or snack or sugar laden or sugar sweeten* or unhealthy) adj1 (beverag* or food$)).ti,id. |
| 27 | "beverages (nonalcoholic)"/ or binge eating/ or calories/ or Diets/ or drinking behavior/ or exp Eating Behavior/ or eating attitudes/ or food deprivation/ or fast food/ or exp Food/ or Food Intake/ or food preferences/ or food preparation/ or food safety/ or hunger/ or meat consumption/ or exp Nutrition/ or (exp Supply Chains/ and exp Food/) or ((diet* or food$ or eat* or fruit$ or meal$ or produce$ or vegetable$) adj3 (choice$ or choos* or habit$ or option$ or prefer*)).ti,id. or (poor diet* or ((healthy or nutritious or nutrition* or unhealthy or non-nutritious) adj1 (diet$ or food$ or ingredient$ or meal$))).ti,id. |
| 28 | (cooking or candy or artificially sweetened beverages or soft drink* or ((consum* or diet* or eat* or intake) adj2 (fat or high-fat)) or fruit$ or fast food$ or nutrition or overnutrition or salad or snacks or snacking or vegetable$).ti. |
| 29 | ((lacking or "lack of" or "no" or proximity or without) adj3 (bakery or bread or butcher shop or deli or grocer* or food retail* or food seller* or food store* or food vendor$ or fruit$ or health$ food$ or nutritious food$ or produce or supermarket$ or vegetable$)).ti,id. or ((lacking or "lack of" or "no" or without) adj3 (bakery or bread or butcher shop or deli or grocer* or food retail* or food seller* or food store* or food vendor$ or fruit$ or health$ food$ or nutritious food$ or produce or supermarket$ or vegetable$)).ab. /freq=2 |
| 30 | 19 and (or/26-29) |
| 31 | healthy eating/ |
| 32 | (((balanced or calories or low calory or healthy or recommend* or fat or fats or fiber* or low-fat) adj1 diet*) or (nutritional adj1 (require* or status)) or ((cook* or eat* or meal$ or diet$) adj3 (fruit or high fiber or green$ or lean* meat$ or low fat or low sodium or produce or salad$ or vegetablle$ or whole grain$))).ti,id. |
| 33 | ((exp Policy Making/ and exp Nutrition/) or ((diet* or eating or food or nutrition*) adj2 (guide* or reccomend*)).ti,id.) and (Compliance/ or (adher* or comply*).ti,id.) |
| 34 | (barrier$ or hurdle$ or impediment$ or obstacle$).ti. |
| 35 | Transportation/ or Public Transportation/ or ((insecur* or lacking or "lack of") adj3 (automobile$ or car$ or bus* or travel* or transport* or vechicle$)).ti,id. |
| 36 | behavioral economics/ or economic disadvantage/ or poverty/ or (pricing or price or ((diet or food or grocer* or produce or vegetable$) adj2 cost$)).ti. |
| 37 | income level/ or disadvantaged/ or economic inequality/ or economic resources/ or "income (economic)"/ or poverty/ |
| 38 | Financial Literacy/ or Literacy/ or Information Literacy/ or Health Literacy/ or health knowledge/ or self-efficacy/ or ((cooking or diet* or food or health or nutrition*) adj2 (illit* or litera or knowledge or skill)).ti,id. |
| 39 | (or/31-32) and (or/33-38) [barriers to nutritious food] |
| 40 | ((automobile* or bus or car or fare or taxi or transit or transport*) adj3 (grocer* or food)).ti,id. or ((automobile* or bus or car or fare or taxi or transit or transport*) adj3 (grocer* or food)).ab. |
| 41 | ((commut* or distance$ or distant or far or far away or time or travel* or barrier$ or hurdle$ or impediment* or mobility or obstacle$) adj3 (grocer* or food or supermarket$)).ti,id,ab. |
| 42 | ((commut* or distance$ or distant or far or far away or time or travel* or barrier$ or hurdle$ or impediment* or mobility or obstacle$) adj3 ((convenienc* or corner*) adj1 store*)).ti,id,ab. |
| 43 | ((commut* or distance$ or distant or far or far away or travel* or barrier$ or hurdle$ or impediment* or mobility or obstacle$) adj5 (bread or butcher$ or dairy or deli or delicatessen or grocer* or food or fruit$ or milk or produce or vegetable$)).mp. |
| 44 | or/39-43 [all lbarriers to a healthy diet] |
| 45 | 8 or 9 or 14 or 15 or 18 or 24 or 25 or 30 or 44 |
| 46 | limit 45 to (100 childhood or 160 preschool age or 180 school age or 200 adolescence or 320 young adulthood ) |
| 47 | preschool students/ or nursery school students/ |
| 48 | pediatrics/ |
| 49 | (adolescent* or ((before or prior to preceding) adj3 (adulthood or maturity)) or child or children or early years or (early adj2 li?e$) or emerging adult$ or highschooler* or highschool age* or high school age* or preschooler* or pre-schooler$ or pediatric* or school age* or teen$ or teen-age* or teenage* or toddler$ or young adult*).ti,id,hw. |
| 50 | (("2" or "3" or "4" or "5" or "6" or "7" or "8" or "9" or "10" or "11" or "12" or "13" or "14" or "15" or "16" or "17" or "18" or "19" or "20" or "21") adj2 (age$ or year$ old$)).ti,id. |
| 51 | 47 or 48 or 49 or 50 |
| 52 | 45 and 51 |
| 53 | 46 or 52 |
| 54 | limit 53 to yr="2013 -Current" |

**Appendix 2: Quality Assessments of Included Studies**

**Table A2a: Studies assessed using JBI Checklist for Analytical Cross-Sectional Studies**

| Study | JBI Checklist for Analytical Cross Sectional Studies | | | | | | | | | |
| --- | --- | --- | --- | --- | --- | --- | --- | --- | --- | --- |
|  | 1. Clear inclusion criteria | 2. Subjects and setting described | 3. Valid and reliable exposure measurement | 4. Objective, standard condition measurement | 5. Confounding factors identified | 6. Strategies for confounding factors | 7. Valid and reliable outcome measurement | 8. Appropriate statistical analysis | Overall score* | Rating^†^ |
| Anderson, 2023 ^(30)^ | Yes | No | Yes | Yes | Yes | Yes | Yes | Yes | 7 | High |
| Angelopoulou, 2019 ^(80)^ | Yes | Yes | Yes | No | No | No | Yes | No | 4 | Moderate |
| Arnaud, 2018 ^(57)^ | Yes | Yes | Yes | Yes | Yes | No | Yes | Yes | 7 | High |
| Bahanan, 2021 ^(64)^ | Yes | No | Yes | Yes | Yes | Yes | Yes | Yes | 7 | High |
| Burke, 2016 ^(65)^ | Yes | Yes | Yes | No | Yes | Yes | Yes | Yes | 7 | High |
| Canter, 2017 ^(81)^ | No | Yes | Yes | Yes | No | Yes | No | Yes | 5 | Moderate |
| Cassidy-Vu, 2022 ^(66)^ | No | Yes | Yes | Yes | Yes | Yes | Yes | Yes | 7 | High |
| Dong, 2023 ^(59)^ | Yes | Yes | Yes | No | Yes | Yes | Yes | Yes | 7 | High |
| Drennan, 2019 ^(82)^ | Yes | Yes | Yes | No | Yes | Yes | No | Yes | 6 | Moderate |
| Edwards, 2023 ^(67)^ | Yes | Yes | Yes | No | Yes | Yes | Yes | Yes | 7 | High |
| Fulay, 2022 ^(68)^ | Yes | No | Yes | Yes | Yes | Yes | Yes | Yes | 7 | High |
| Godrich, 2019 ^(55)^ | Yes | Yes | Yes | No | Yes | Yes | No | Yes | 6 | Moderate |
| Hill, 2020 ^(83)^ | No | Yes | No | No | Yes | Yes | Yes | Yes | 5 | Moderate |
| Hutchinson, 2022 ^(56)^ | Yes | No | Yes | Yes | Yes | Yes | Yes | Yes | 7 | High |
| Jun, 2021 ^(84)^ | Yes | Yes | Yes | Yes | Yes | No | Yes | No | 6 | Moderate |
| Jung, 2023 ^(58)^ | No | Yes | No | No | No | No | No | No | 1 | Low |
| Lee, 2019a ^(69)^ | Yes | Yes | Yes | Yes | Yes | Yes | Yes | Yes | 8 | High |
| Lee, 2019b ^(70)^ | Yes | Yes | Yes | No | Yes | Yes | Yes | Yes | 7 | High |
| Maldonado, 2022 ^(60)^ | Yes | Yes | Yes | Yes | Yes | Yes | Yes | Yes | 8 | High |
| Mangini, 2015 ^(62)^ | Yes | Yes | Yes | No | Yes | Yes | No | Yes | 6 | Moderate |
| Maynard, 2019 ^(100)^ | Yes | Yes | Yes | Yes | Yes | Yes | No | Yes | 7 | High |
| Na, 2020 ^(72)^ | Yes | Yes | Yes | Yes | Yes | Yes | No | Yes | 7 | High |
| Pirkle, 2014 ^(51)^ | Yes | Yes | Yes | Yes | Yes | Yes | Yes | Yes | 8 | High |
| Poole-Di Salvo, 2016 ^(73)^ | Yes | Yes | Yes | No | Yes | Yes | Yes | Yes | 7 | High |
| Rossen, 2016 ^(74)^ | Yes | Yes | Yes | Yes | Yes | Yes | Yes | Yes | 8 | High |
| South, 2019 ^(75)^ | Yes | No | Yes | Yes | Yes | Yes | Yes | Yes | 7 | High |
| Tarullo, 2020 ^(76)^ | Yes | Yes | Yes | No | Yes | Yes | Yes | Yes | 7 | High |
| Tester, 2016 ^(77)^ | Yes | No | Yes | Yes | Yes | Yes | Yes | Yes | 7 | High |
| Thomas, 2019 ^(101)^ | Yes | Yes | Yes | No | Yes | Yes | Yes | Yes | 7 | High |
| Trude, 2022 ^(79)^ | Yes | Yes | Yes | No | Yes | Yes | Yes | Yes | 7 | High |

*Only items that received a “Yes” were counted toward the overall score.

^†^Ratings were based on the total criteria met by the study (1-3 Low; 4-6 Moderate, 7-8 High).

**Table A2b: Studies assessed using JBI Checklist for Cohort Studies**

| Study | JBI Checklist for Cohort Studies | | | | | | | | | | | | |
| --- | --- | --- | --- | --- | --- | --- | --- | --- | --- | --- | --- | --- | --- |
|  | 1. Groups similar | 2. Exposures measured consistently | 3. Valid and reliable exposure measurement | 4. Confounding factors identified | 5. Strategies for confounding factors | 6. Participants free of outcome at study start | 7. Valid and reliable outcome measurement | 8. Adequate follow-up time | 9. Follow up complete | 10. Strategies for incomplete follow up | 11. Appropriate statistical analysis | Overall score* | Rating^†^ |
| Clemens, 2021a ^(53)^ | Yes | Yes | Yes | Yes | Yes | Yes | Yes | Yes | No | No | Yes | 9 | High |
| Clemens, 2021b ^(54)^ | Yes | Yes | Yes | Yes | Yes | Yes | Yes | Yes | No | No | Yes | 9 | High |
| Mangini, 2018 ^(85)^ | Yes | Yes | Yes | Yes | Yes | Yes | Yes | Yes | No | No | Yes | 9 | High |
| Men, 2021 ^(29)^ | Yes | Yes | No | Yes | Yes | Yes | Yes | Yes | No | No | Yes | 8 | Moderate |
| Metallinos-Katsaras, 2016 ^(61)^ | Yes | Yes | Yes | Yes | Yes | Yes | Yes | Yes | No | No | Yes | 9 | High |
| Sandoval, 2021 ^(63)^ | Yes | Yes | Yes | Yes | No | Yes | Yes | Yes | Yes | No | Yes | 9 | High |

*Only items that received a “Yes” were counted toward the overall score.

^†^Ratings were based on the total criteria met by the study (1-4 Low; 5-8 Moderate, 9-11 High).
